# PAK6 promotes homologous-recombination to enhance chemoresistance to oxaliplatin through ATR/CHK1 signaling in gastric cancer

**DOI:** 10.1038/s41419-022-05118-8

**Published:** 2022-07-28

**Authors:** Weicai Huang, Zhen Han, Zepang Sun, Hao Feng, Liying Zhao, Qingyu Yuan, Chuanli Chen, Shitong Yu, Yanfeng Hu, Jiang Yu, Hao Liu, Guoxin Li, Yuming Jiang

**Affiliations:** 1 Department of General Surgery & Guangdong Provincial Key Laboratory of Precision Medicine for Gastrointestinal Tumor, Nanfang Hospital, The First School of Clinical Medicine, Southern Medical University, Guangzhou, Guangdong 510515 China; 2 Department of Medical Imaging Center, Nanfang Hospital, Southern Medical University, No. 1838, Guangzhou Avenue North, 510515 Guangzhou, China

**Keywords:** Gastric cancer, Cancer therapeutic resistance, Chemotherapy, Tumour biomarkers

## Abstract

Chemoresistance remains the primary challenge of clinical treatment of gastric cancer (GC), making the biomarkers of chemoresistance crucial for treatment decision. Our previous study has reported that p21-actived kinase 6 (PAK6) is a prognostic factor for selecting which patients with GC are resistant to 5-fluorouracil/oxaliplatin chemotherapy. However, the mechanistic role of PAK6 in chemosensitivity remains unknown. The present study identified PAK6 as an important modulator of the DNA damage response (DDR) and chemosensitivity in GC. Analysis of specimens from patients revealed significant associations between the expression of PAK6 and poorer stages, deeper invasion, more lymph node metastases, higher recurrence rates, and resistance to oxaliplatin. Cells exhibited chemosensitivity to oxaliplatin after knockdown of PAK6, but showed more resistant to oxaliplatin when overexpressing PAK6. Functionally, PAK6 mediates cancer chemoresistance by enhancing homologous recombination (HR) to facilitate the DNA double-strand break repair. Mechanistically, PAK6 moves into nucleus to promote the activation of ATR, thereby further activating downstream repair protein CHK1 and recruiting RAD51 from cytoplasm to the DNA damaged site to repair the broken DNA in GC. Activation of ATR is the necessary step for PAK6 mediated HR repair to protect GC cells from oxaliplatin-induced apoptosis, and ATR inhibitor (AZD6738) could block the PAK6-mediated HR repair, thereby reversing the resistance to oxaliplatin and even promoting the sensitivity to oxaliplatin regardless of high expression of PAK6. In conclusion, these findings indicate a novel regulatory mechanism of PAK6 in modulating the DDR and chemoresistance in GC and provide a reversal suggestion in clinical decision.

## Introduction

Gastric cancer (GC) is one of the most common malignant tumors and the fourth leading cause of cancer-related deaths in the world [1, 2]. Curative resection is the primary treatment option for patient with GC. However, most of the patients are diagnosed at advanced stages of the disease with local or distant metastases due to atypical and occult symptoms of the early stages, leading to poor prognosis or recurrence [3]. Oxaliplatin is one of the most effective antitumor drugs for treatment of advanced patients with GC [4]. It can form platinum-DNA adducts that block DNA replication and transcription, thereby leading to substantial DNA double-strand breaks (DSBs) as well as cell apoptosis [5]. Although the treatment landscape of advanced gastric cancer has dramatically changed by oxaliplatin-based chemotherapy and targeted therapy with a significant improvement in overall survival (OS), the 5-year rates of OS for advanced GC remains limited and most of the patients eventually experience chemoresistance which is a common cause of the treatment failure [6]. Therefore, it is crucial to find an innovative predictor of chemotherapy resistance and further converse resistance to improve treatment outcomes for advanced GC.

P21-actived kinase 6 (PAK6) gene is a key member of a family of class II p21-stimulated serine/threonine protein kinases [7]. Containing a carboxyl-terminal kinase domain and an amino-terminal Cdc43/Rac interactive binding domain, the PAK6 protein participates in multiple cellular functions including gene transcription, cytoskeleton formation, cell motility, drug resistance, and cell apoptosis [8–11]. Several previous studies have evaluated the modulation of cellular progress by PAK6 and the important role of PAK6 to modulate the chemosensitivity. According to Lin et al., PAK6 causes therapy resistance to tyrosine kinase inhibitors in chronic myeloid leukemia through miR-185/PAK6 axis [12]. Elsewhere, a study conducted by Wen et al. showed that knockdown PAK6 can enhance chemosensitivity to docetaxel in prostate cancer [13]. In addition, our previous study also found that PAK6 increases chemoresistance to 5-FU/oxaliplatin chemotherapy in GC [14]. However, the molecular mechanism of PAK6 in modulating chemoresistance to oxaliplatin in GC still remains elusive.

The present study identified that PAK6 mainly influence chemoresistance towards oxaliplatin by enhancing the homologous recombination (HR) mediated DNA repair. With the immunohistochemical specimens of GC and treatment information, it was found that high expression of PAK6 leads to poor prognosis in the patients treated with oxaliplatin after surgery but not those threated through surgery only or capecitabine (an oral pro drug of 5-fluorouracil) after surgery. It was evident that knockdown of PAK6 increased sensitivity to oxaliplatin, whereas overexpression of PAK6 promoted the resistance to oxaliplatin.

Moreover, research studies on the mechanism showed that PAK6 rapidly move into nucleus to activate ATR, thereby further activating CHK1 and recruiting RAD51 from cytoplasm to the DNA damage site in nucleus and hence promote the HR repair in respond to oxaliplatin. For the first time the present study revealed that PAK6 is a predictive biomarker of chemoresistance to oxaliplatin in patients with GC. Further, it was found that the PAK6-mediated HR DNA damage repair can be reversed by adding ATR inhibitor on the basis of oxaliplatin to block the phosphorylation of ATR and CHK1. Therefore, this may provide an early warning of clinical deterioration and a further treatment suggestion for advanced GC patients.

## Results

### PAK6 expression is correlated with poor prognosis and the therapeutic efficacy of oxaliplatin in GC

**Fig. 1:**
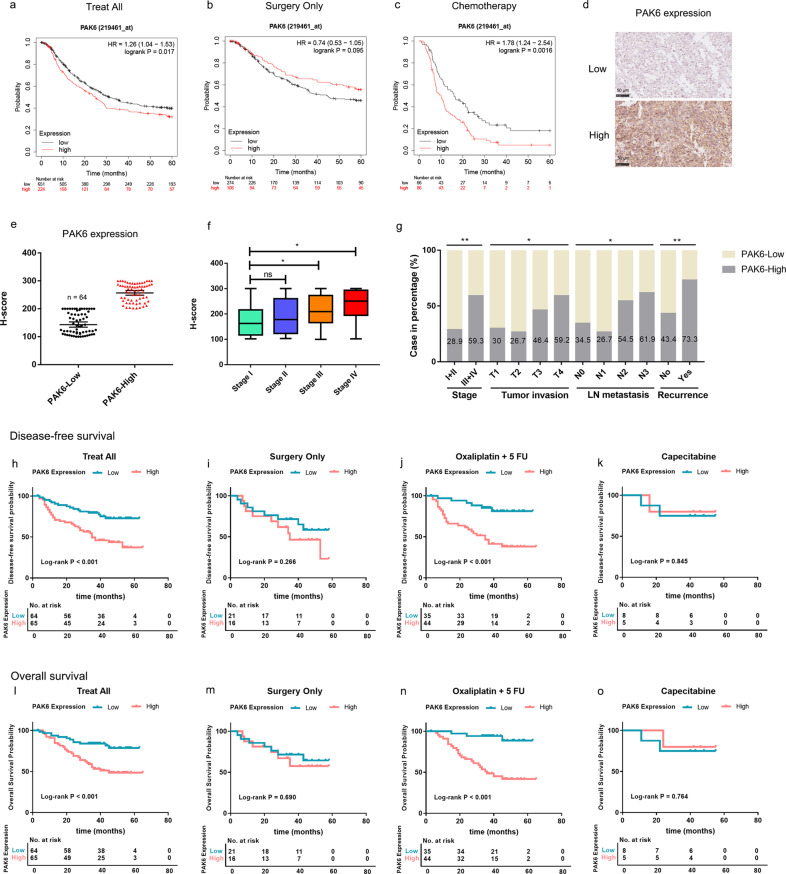
PAK6 is associated with poor prognosis and the therapeutic efficacy of oxaliplatin in GC. **a** Kaplan–Meier survival curves of GC patients with PAK6 expression in the KM-Plotter database (www.kmplot.com). **b** Subgroup survival analysis according to the PAK6 expression with GC patients treated with surgery only in the KM-Plotter database. **c** Subgroup survival analysis of PAK6 expression with GC patients treated with chemotherapy in the KM-Plotter database. **d** Representative graphs of PAK6 expression (high expression and low expression) in the tumor tissues. **e** Quantification of the immunohistochemical staining intensity of the PAK6 expression in GC patients. **f** Quantification of immunohistochemical staining intensity of the PAK6 expression in different TNM stages. **g** Percentage of GC patients with high PAK6 expression in the groups classified by TNM stage, tumor invasion, lymph node metastasis, and recurrence. **h** Kaplan–Meier survival curves of disease-free survival of the GC patients according to the PAK6 expression. **i** Subgroup survival analysis of disease-free survival of GC patients treated with surgery only according to the PAK6 expression. **j** Subgroup survival analysis of disease-free survival of GC patients treated with chemotherapy of oxaliplatin/5 FU after surgery according to the PAK6 expression. **k** Subgroup survival analysis of disease-free survival of GC patients treated with chemotherapy of capecitabine after surgery according to the PAK6 expression. **l** Kaplan–Meier survival curves of overall survival of the GC patients according to the PAK6 expression. **m** Subgroup survival analysis of overall survival of GC patients treated with surgery only according to the PAK6 expression. **n** Subgroup survival analysis of overall survival of GC patients treated with chemotherapy of oxaliplatin/5 FU after surgery according to the PAK6 expression. **o** Subgroup survival analysis of overall survival of GC patients treated with chemotherapy of capecitabine after surgery according to the PAK6 expression. *: *P* < 0.05, **: *P* < 0.01, ns: no statistical difference.

To investigate the clinical role of PAK6 in GC, the public online database KM plotter (http://Kmplot.com/analysis/) was used to evaluate the prognostic values of PAK6 in gastric cancer. The 5-years OS analysis of PAK6 identified that high expression of PAK6 was significantly positively associated with poor prognosis (*P* = 0.017, Fig. 1a). When performing subgroup analysis, it was noted that the high expression of PAK6 was negatively associated with the survival rate of the patients who were treated with surgery only (*P* = 0.095, Fig. 1b), but strongly correlated with poor prognosis of patients who were treated with surgery followed by chemotherapy (*P* = 0.002, Fig. 1c), indicating PAK6 might modulate drug sensitivity in gastric cancer.

There were 129 GC patients from our hospital that were included in this study, and the patients were classified into PAK6-High group and PAK6-Low group according to the expression level of PAK6 of tumor tissues (Fig. 1d, e). Clinical data analysis revealed that high expression of PAK6 resulted in later stage (stage III and IV), deeper invasion, more lymph nodes metastases, and higher recurrence rate (Fig. 1f, g, and Table S1). Survival analysis showed that, high expression of PAK6 was significantly associated with poor prognosis of disease-free survival (DFS) and OS (Fig. 1h, l). It was also found that PAK6 expression had no effect on survival of the patients who were treated with surgery only, but showed significant unfavorable prognosis of the patients who were treated with chemotherapy of oxaliplatin plus 5-FU after surgery which was consistent with the results of the public online dataset (Fig. 1i, j, m and n). The potential effect of PAK6 expression on the prognosis of patients who were treated with capecitabine was also assessed in the present study. However, it was found that there was no significant difference in the effect of PAK6 expression on prognosis of the patients (Fig. 1k, o). Therefore, results of the present study supported PAK6 as a significant predictor for the therapeutic efficacy of oxaliplatin in GC, but not of drug of 5-FU.

### PAK6 reduces the cytotoxicity of oxaliplatin in gastric cancer cell lines

**Fig. 2:**
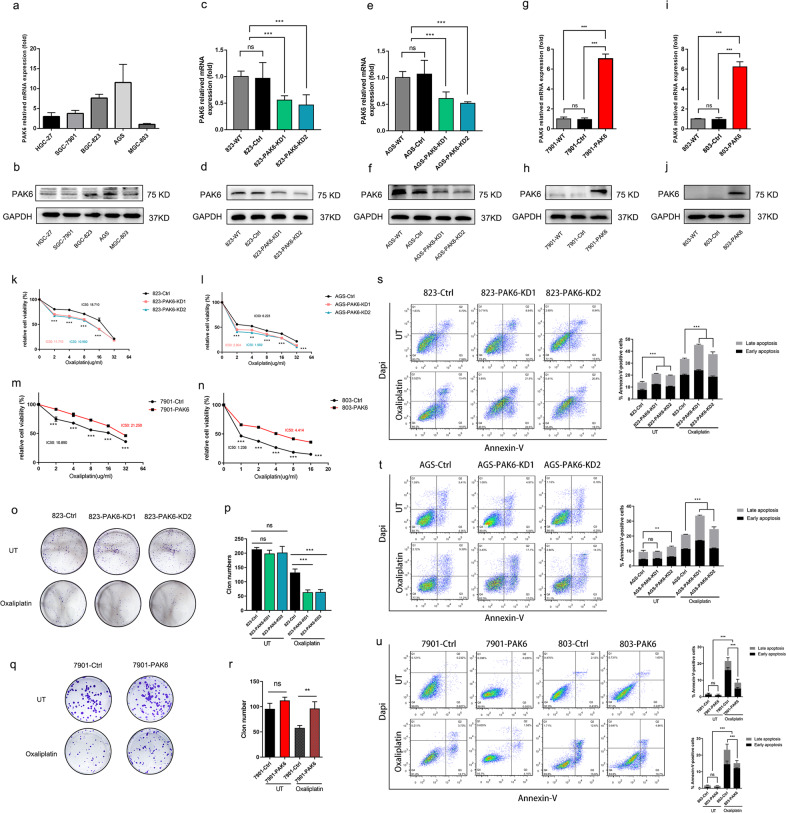
PAK6 increases the chemoresistance to oxaliplatin in GC cells. **a**, **b** The expression of PAK6 in HGC-27, SGC-7901, BGC-823, AGS, and MGC-803 GC cell lines. **c**–**f** Knock down of the expression of PAK6 in 823 (**c**, **d**) and AGS (**e**, **f**) GC cells. **g**–**j** Overexpressing the expression of PAK6 in 7901 (**g**, **h**) and 803 (**i**, **j**) GC cells. **k**–**n** Dose-response curves of control cells or PAK6 knocked down cells in 823 (**k**) and AGS (**l**) GC cells after treated with oxaliplatin for 24 h. The IC_50_ of the oxaliplatin in the 823 cell line are 18.701(823-Ctrl cells), 11.710 (823-PAK6-KD1 cells), and 10.960 (823-AK6-KD2 cells) μg/ml. The IC_50_ of the oxaliplatin in the AGS cell line are 6.233 (AGS-Ctrl cells), 2.904 (AGS-PAK6-KD1 cells), and 1.969 (AGS-PAK6-KD2 cells) μg/ml. Dose-response curves of control cells or PAK6 overexpression cells in 7901 (**m**) and 803 (**n**) GC cells after treated with oxaliplatin for 24 h. The IC_50_ of the oxaliplatin in the cells are 10.890 (7901-Ctrl cells), 21.250 (7901-PAK6 cells), 1.236 (803-Ctrl cells), and 4.414 (803-PAK6 cells) μg/ml. **o**–**r** Colony formation ability of PAK6 knocked down cells in 823 GC cell line (**o**, **p**) and PAK6 overexpression cells in 7901 (**q**, **r**) GC cell line treated with or without oxaliplatin (0.3 μg/ml in 823 cells or 0.5 μg/ml in 7901 cells). **s**, **t** Apoptosis analysis of control cells and PAK6 knocked down cells in 823 and AGS GC cells treated with or without oxaliplatin. **u** Apoptosis analysis of control cells and PAK6 overexpression cells in 7901 and 803 GC cells treated with or without oxaliplatin. Data presented as mean ± SD of three independent replicates. *: *P* < 0.05, **: *P* < 0.01, ***: *P* < 0.001, ns: no statistical difference.

To further investigate the intrinsic relationship between PAK6 expression level and the antitumor efficacy of oxaliplatin, the raw expression levels of PAK6 of GC cells were firstly detected (Fig. 2a, b). Then the PAK6 was knocked down in BGC-823 as well as AGC cells, and was overexpressed in SGC-7901 as well as MGC-803 cells (Fig. 2c–j). It was found that the knockdown of PAK6 led to a substantial decrease in the half maximal inhibitory concentration (IC_50_) of oxaliplatin (*P* < 0.001) in the 823 and AGS cells. On the contrary, it was found that the overexpression of PAK6 in 7901 and 803 cells significantly promoted the chemoresistance to oxaliplatin (*P* < 0.001, Fig. 2k–n). However, except AGS PAK6 knockdown cells showed a slight decrease in the IC_50_ of 5-FU, no significant difference was found in other cells (Fig. S1a–d).

Results of the clonogenic assay showed a strong growth inhibition after being exposed to oxaliplatin in AGS and 823 cells (*P* < 0.001, Fig. 2o, p, and Fig. S2a, b). While the overexpression of PAK6 showed remarkable increase in chemoresistance of oxaliplatin by maintaining the cell proliferate as compared with control cells in the 7901 and 803 cells (*P* < 0.01, Fig. 2q, r, and Fig. S2c, d). Additionally, flow cytometry analysis reflected that the knockdown of PAK6 resulted in severer apoptosis when treated with oxaliplatin (*P* < 0.001, Fig. 2s, t). Moreover, it was evident that the overexpression of PAK6 significantly reduced oxaliplatin-induced apoptosis in 7901 and 803 cells. (*P* < 0.001, Fig. 2u). Overall, results of the present study suggested that PAK6 is positively correlated with the chemoresistance to oxaliplatin and can reduce the cytotoxicity of oxaliplatin in GC cell lines.

### PAK6 promotes oxaliplatin resistance of GC cells by enhancing DNA damage repair

**Fig. 3:**
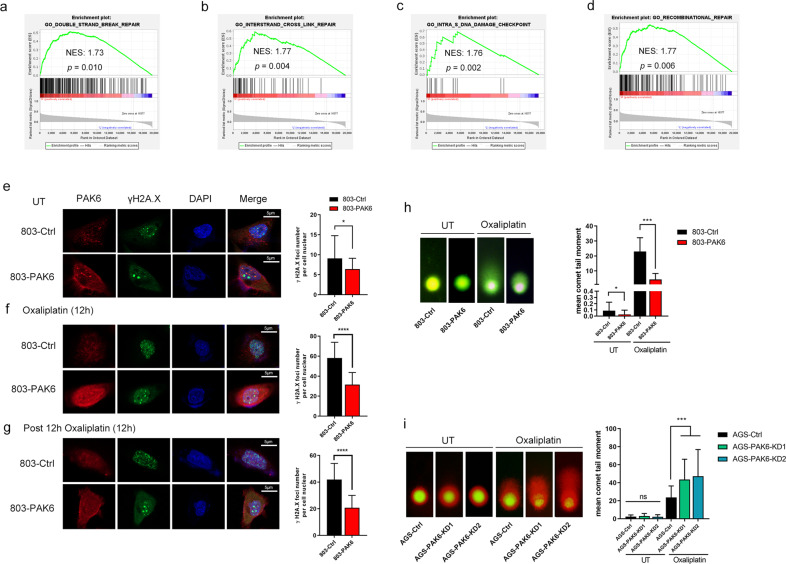
PAK6 reduces DNA damage induced by oxaliplatin in GC cells. **a**–**d** Go analysis of the gene pathways differentially expressed between PAK6-high expression and PAK6-low expression gastric cancer samples in TCGA database. Four representative GSEA-enrichment plots were shown. **e** Left: PAK6 staining (red), γH2A.X staining (green), and DAPI staining (blue) in the control cells and PAK6 overexpression cells in 803 GC cell line; Right: Quantification of mean γH2A.X foci per cell. **f** Left: PAK6 staining (red), γH2A.X staining (green), and DAPI staining (blue) in the control cells and PAK6 overexpression cells in 803 GC cell line after treated with oxaliplatin for 12 h; Right: Quantification of mean γH2A.X foci per cell. **g** Left: PAK6 staining (red), γH2A.X staining (green), and DAPI staining (blue) in the control cells and PAK6 overexpression cells in 803 GC cell line after recovery from oxaliplatin treating (12 h) for 12 h; Right: Quantification of mean γH2A.X foci per cell. At least 30 cells per group were included for the counting and quantification. **h**, **i** Left: Representative images of neutral comet assays of control cells, PAK6 overexpression cells, and PAK6 knocked down cells in the absence and presence of oxaliplatin; Right: Quantification of the percentages of DNA tail moments. At least 30 cells per group were included for the counting and quantification. UT: untreated. *: *P* < 0.05, ***: *P* < 0.001, ****: *P* < 0.0001, ns: no statistical difference.

To investigate the potential mechanisms of PAK6 in resisting oxaliplatin-induced cytotoxicity, the GSEA was performed to assess the regulatory pathway using TCGA dataset regarding gastric cancer. Notably, results of the GSEA showed significant enrichment in DNA damage response (DDR) gene sets such as DSB repair and inter-strand cross link repair (Fig. 3a–d). Since γH2A.X is a major marker of DNA damage [15], the γH2A.X foci number in nucleus of cells was also detected to evaluate the DNA damage level of the cells in the absence and presence of oxaliplatin. Immunofluorescence results showed that the overexpression of PAK6 in 7901 and 803 cells caused a reduce of γH2A.X foci in the stimulation of oxaliplatin for 12 h compared with the control cells (Fig. 3e, f and Fig. S3a, b). Moreover, when removing the oxaliplatin after a 12 h treatment and replacing it with fresh media for 12 h, the γH2A.X foci number in PAK6 overexpression cells was rapidly reduced into a low level whereas foci number in the control cells was still significantly maintained in a high level, indicating a persistent DNA damage in the control cells (Fig. 3g and Fig. S3c).

Additionally, a comet assay was also performed to further assess the potential role of PAK6 in DDR after exposure to oxaliplatin. Expectedly, it was noted that the overexpression of PAK6 in 7901 and 803 cells significantly enhanced the repair efficacy after the exposure to oxaliplatin (Fig. 3h and Fig. S3d). By comparison, the PAK6 knockdown cells in AGS cells and 823 cells showed severe repair defect after oxaliplatin exposure (Fig. 3i and Fig. S3e). Therefore, it was evident that PAK6 has a vital capacity of promoting DNA damage repair to improve the chemoresistance to oxaliplatin in GC cells.

### PAK6 enhances DNA HR repair via promoting ATR/CHK1 signaling activation in nucleus

**Fig. 4:**
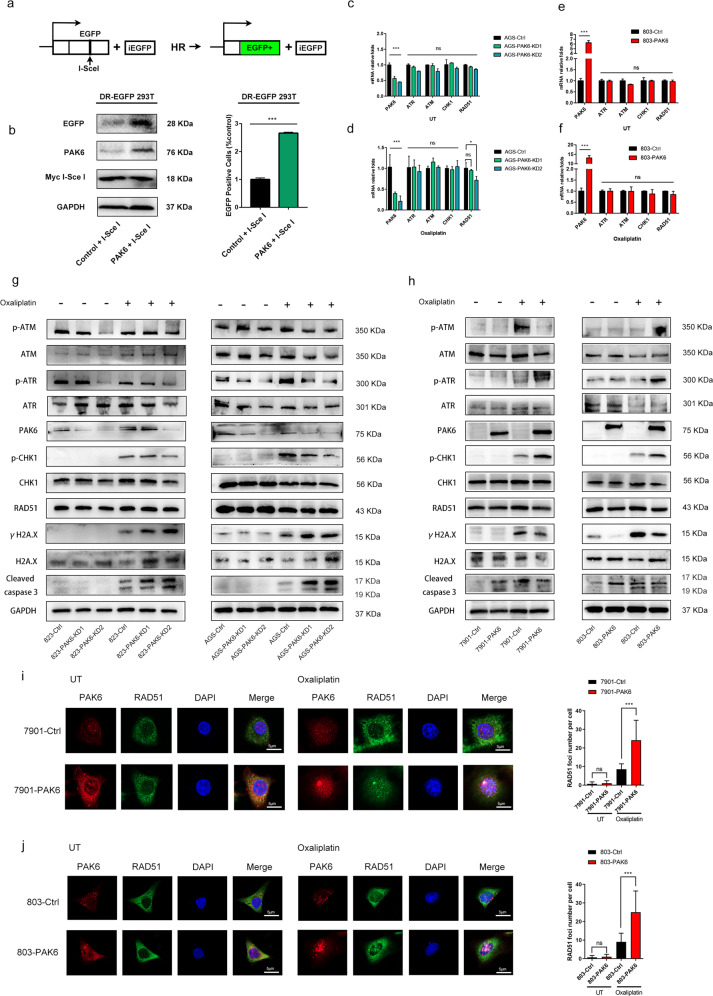
PAK6 regulates the efficiency of HR. **a** Schematic illustration of HR in HEK 293T-HR-EGFP reporter cells. **b** PAK6 overexpression leads to increase HR efficiency. HEK 293T-HR-EGFP cells were cotransfected with either empty controls or PAK6 and Myc-I-SceI plasmids. The expression of EGFP, PAK6, and Myc-I-SceI was detected by WB analysis. The percentage of EGFP positive cells were examined by flow cytometry. **c**–**f** Real-time PCR analysis of the mRNA level of the key molecules involved in HR pathway in PAK6 knocked down and overexpression cells in the absence (**c**, **e**) and presence (**d**, **f**) of oxaliplatin. Data presented as mean ± SD of three independent replicates. **g**, **h** WB analysis of HR related key genes, PAK6, γH2A.X, cleaved caspase 3, and GAPDH in PAK6 knocked down cells of 823 and AGS GC cell lines (**g**) and PAK6 overexpression cells of 7901 and 803 GC cell lines (**h**) in the absence and presence of oxaliplatin. **i**, **j** Left: Representative images of PAK6 staining (red), RAD51 staining (green), and DAPI staining (blue) of control cells and PAK6 overexpression cells in 7901 (**i**) and 803 (**j**) GC cell lines in the absence and presence of oxaliplatin. Right: Quantification of mean γH2A.X foci per cell. At least 30 cells per group were included for the counting and quantification. *: *P* < 0.05, ***: *P* < 0.001, ns: no statistical difference.

HR repair and non-homologous end joining (NHEJ) are the main repair pathways of DSB. The two are switched in different cell-cycle stage. In G0/G1 stage, DNA damage repair factor 53BP1 opposes the resection of the damaged DNA, thereby promoting NHEJ, an error-prone repair pathway, and inhibit HR at the same time [16]. In S-G2 stage, ATR and CHK1 control the G2/M checkpoint thereby opposes the NHEJ and enhance the error-free HR basing on the template of intact sister chromatid [17]. Increasing studies have revealed that HR is the main pathway involved in DNA repair after treatment of anti-tumor agents [18]. Therefore, the present study hypothesized whether PAK6 affects the HR repair to improve the chemoresistance to oxaliplatin. Notably, cell cycle analysis showed that the G2/M cell cycle arrest in PAK6 overexpression cells followed by the treatment of oxaliplatin in 803 cells, indicating PAK6 might mainly affect HR repair (Fig. S4a–f). Further, the finding was further validated through plasmid-based HR repair reporter assay on HEK-293T cells. In this study, I-SceI plasmid was transfected into 293T-HR-EGFP-PAK6 cells and 293T-HR-EGFP-Vec cells to induce DSBs. Enhanced green fluorescent protein would be expressed if the damaged DNA was repaired by HR (Fig. 4a). Expectedly, overexpressing the PAK6 showed a dramatical increased ratio of EGFP positive cells and high level of EGFP (Fig. 4b), which supported the fact that PAK6 could regulate HR efficacy to affect the sensitivity to oxaliplatin in GC cells.

Further, the expressions of key regulating genes of HR were detected. However, it was found that although the RAD51 was downregulated in PAK6 knockdown cells in AGS, neither the mRNA nor protein levels of ATR, ATM, CHK1 or RAD51 were significantly changed in the PAK6 knockdown cells or overexpressing cells (Fig. 4c–h). RNA-sequencing was also carried to detect the key DDR genes, and similarly, the results showed that genes including ATR, ATM, CHK1 and RAD51 were not significantly affected by PAK6 (Fig. S5a–d). Notably, the protein levels of DNA damage markers including γH2A.X and cleaved caspase-3 were increased after PAK6 knockdown in 823 and AGS cells whereas it was decreased following PAK6 overexpressing in 7901 and 803 cells after oxaliplatin exposure (Fig. 4g, h).

While the phosphorylated protein levels of HR efforts such as p-ATR and p-CHK1 were decreased in the PAK6 knockdown cells and increased in the PAK6 overexpressing cells after oxaliplatin exposure (Fig. 4g, h). This indicated that PAK6 affected HR pathway to regulate the cell survival. Since the RAD51 is an important effector protein in the process of ATR-mediated HR, and the nucleus is the central site for RAD51 to perform HR repair, the current study also evaluated whether PAK6 mediated the RAD51 recruitment from cytoplasm to nucleus to enhance DNA repair. Therefore, immunofluorescence assay was performed to investigate the correlation between PAK6 and RAD51. Expectedly, results revealed that RAD51 was increased dramatically in nucleus of PAK6 overexpressed cells after the treatment with oxaliplatin (Fig. 4i, j). Therefore, it was indicated that PAK6 can significantly enhance the recruitment of RAD51 to nucleus during the process of HR repair.

### ATR/CHK1 signaling activation is necessary for PAK6-mediated HR repair

Since ATR signaling promotes the RAD51 foci formation to regulate HR repair [19], the present study also hypothesized whether the recruitment of RAD51 by PAK6 is mechanistically dependent on ATR signaling. It was observed that the oxaliplatin-induced phosphorylation of ATR and CHK1 were compromised in cells lacking PAK6 but increased in cells overexpressing PAK6, whereas the oxaliplatin-induced apoptosis protein of γH2A.X and cleaved caspase-3 were increased in cells lacking PAK6 but attenuated in cells overexpressing PAK6 (Fig. 4g–j). This suggested PAK6 may facilitate the activation of ATR and CHK1 after exposure to oxaliplatin and thereby promoting RAD51 foci formation to enhance HR repair as well as reduces cell apoptosis.

**Fig. 5:**
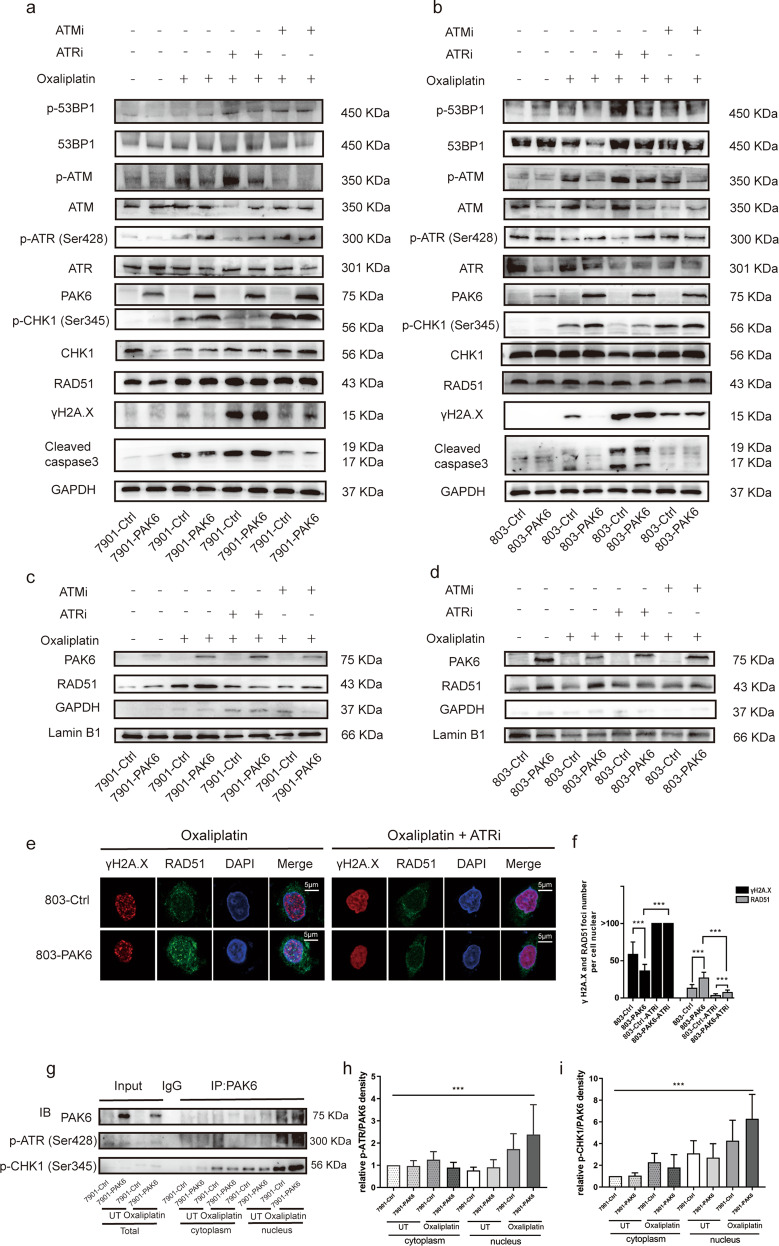
ATR/CHK1 signaling activation is required for PAK6 mediated HR repair. **a**, **b** WB analysis of HR related key genes, PAK6, γH2A.X, cleaved caspase 3, and GAPDH in control cells and PAK6 overexpression cells in 7901 (**a**) and 803 (**b**) GC cell lines. **c**, **d** WB analysis of PAK6, RAD51, GAPDH, and lamin B1 protein in nucleus in control cells and PAK6 overexpression cells in 7901 (**c**) and 803 (**d**) GC cell lines. Cells were untreated, subjected to oxaliplatin, subjected to combination of oxaliplatin and ATR inhibitor AZD6738, or subjected to combination of oxaliplatin and ATM inhibitor AZD0156 for 24 h. **e** Left: Representative images of γH2A.X staining (red), RAD51 staining (green), and DAPI staining (blue) of control cells and PAK6 overexpression cells in 803 GC cell lines in the presence of oxaliplatin. Right: Representative images of γH2A.X staining (red), RAD51 staining (green), and DAPI staining (blue) of control cells and PAK6 overexpression cells in 803 GC cell lines in the presence of combination of oxaliplatin and ATR inhibitor AZD6738. **f** Quantification of mean γH2A.X and RAD51 foci per cell. At least 30 cells per group were included for the counting and quantification. **g** Immunoprecipitation assay with anti-PAK6 antibody from control cells and PAK6 overexpression cells in 7901 GC cell line. Cells were untreated or subjected to oxaliplatin for 24 h. And protein was lysed from cytoplasm and nucleus respectively. **h**, **i** Relative protein band intensity of p-ATR (**h**) and p-CHK1 (**i**) protein normalized against of PAK6. UT: untreated. ATRi: ATR inhibitor AZD6738. ATMi: ATM inhibitor AZD0156. ***: *P* < 0.001.

To investigate this possibility further, the inhibition of ATR and ATM of cells exposed to oxaliplatin was also conducted in the present study. In addition, different concentrations of inhibitors were evaluated to measure the cytotoxicity to eliminate the additional affection of ATR and ATM inhibitors in cell viability (Fig. S6a, b). It was found that AZD6738 effectively abolished the phosphorylation of ATR and its downstream effector kinase p-CHK1, but had no impact on the total expression of PAK6, suggesting that PAK6 may be located in the upstream of ATR and CHK1 in HR signaling (Fig. 5a, b). Notably, it was found that the attenuation of apoptosis protein of γH2A.X and cleaved caspase-3 in PAK6 overexpressing cells were reversed after treated with ATR inhibitor. In PAK6 overexpression cells, the protein level of RAD51 increased by PAK6 in nucleus was dramatically reduced after adding ATR inhibitor as compared with single treatment of oxaliplatin, suggesting that the activation ATR/CHK1 signaling is essential for PAK6 mediated HR repair (Fig. 5c, d). Moreover, IF results showed that, the RAD51 foci formation induced by PAK6 in nucleus was strongly reduced after addition of AZD6738 to oxaliplatin in the 803 cells (Fig. 5e, f). However, the RAD51 foci number in the PAK6 overexpression cells was still higher than the control cells. This was an indication that PAK6 may has the ability to directly promote RAD51 foci formation in the nucleus. Similar result was also found in 7901 cells (Fig. S7a, b).

Since ATM could also activates the phosphorylation of CHK1 [20], the inhibition of ATM was also performed to further detected the alternation of the above proteins. However, it was noted that there was no significant difference (Fig. 5a, b), indicating that the ATM singling is not essential for increased RAD51 in PAK6-mediated HR repair. Moreover, it was found that the interactions between PAK6 with p-ATR and p-CHK1 were strongest in the nucleus after treated with oxaliplatin, whereas the interaction between PAK6 and p-ATR and p-CHK1 were extremely weak in the untreated cells (Fig. 5g–i). Similar trends were also found in the 803 cells (Fig. S7c–e). In general, these results indicated that PAK6 can promote activation of ATR/CHK1 signaling in nucleus, thereby enhancing the HR repair by recruiting RAD51 to nucleus.

### ATR inhibitor AZD6738 combinates with oxaliplatin could re-sensitize PAK6 overexpression GC cells to oxaliplatin via blocking the DNA HR repair

**Fig. 6:**
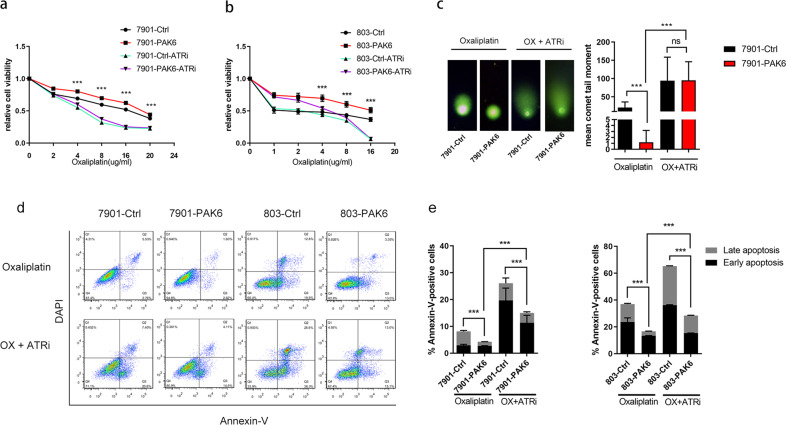
ATR inhibitor AZD6738 combinates with oxaliplatin could re-sensitize PAK6 overexpression GC cells to oxaliplatin. **a**, **b** Dose-response curves of control cells or PAK6 overexpression cells in 7901 (**a**) and 803 (**b**) GC cell line after treated with oxaliplatin or combination of ATR inhibitor AZD6738 and oxaliplatin for 24 h. Data presented as mean ± SD of three independent replicates. **c** Left: Representative images of neutral comet assays of control cells and PAK6 overexpression cells in 7901 GC cell lines in the presence of oxaliplatin or combination of ATR inhibitor AZD6738 and oxaliplatin; Right: Quantification of the percentages of DNA tail moments. At least 30 cells per group were included for the counting and quantification. **d** Apoptosis analysis of control cells and PAK6 overexpression cells in 7901 and 803 GC cell lines in the presence of oxaliplatin or combination of ATR inhibitor AZD6738 and oxaliplatin. **e** The percentages of apoptotic cells were displayed by the bar chart. Data presented as mean ± SD of three independent replicates. ATRi: ATR inhibitor AZD6738. OX: oxaliplatin. *P < 0.05, **P < 0.01, ***P < 0.001, ns: no statistical difference.

Further, cell viability following treatments of oxaliplatin monotherapy and the combination of oxaliplatin and ATR inhibitor was assayed to explore the therapeutic potential of drug combination of ATR inhibitor and oxaliplatin in GC cells, especially in the PAK6 overexpressing cells. Notably, treatment of ATR inhibitor accompany with oxaliplatin re-sensitizes PAK6-induced oxaliplatin-resistant cells to oxaliplatin (Fig. 6a, b). To further understand the mechanism of drug synergy of oxaliplatin and ATR inhibitor in PAK6 overexpressing cells, comet assay was carried out to evaluate the accumulation of DSB and HR repair. Expectedly, catastrophic repair defect was detected in the combination of oxaliplatin and ATR inhibitor AZD6738 (Fig. 6c, Fig. S8) which indicated that the PAK6-induced HR repair against cytotoxicity of oxaliplatin is blocked by ATR inhibitor. Moreover, it was found that the combination of oxaliplatin and ATR inhibitor AZD6738 led to a significant increase in early and late apoptotic cells in PAK6 overexpression cells (Fig. 6d, e). Taken together, it was evident that the combination of oxaliplatin and ATR inhibitor AZD6738 reverses the chemoresistance induced by PAK6 through blocking the PAK6-induced HR repair as well as causing catastrophic DSB and ultimately leading to cell death by apoptosis.

### Utility of the combination of oxaliplatin and ATR inhibitor (AZD6738) enhances the antitumor efficacy in vivo

**Fig. 7:**
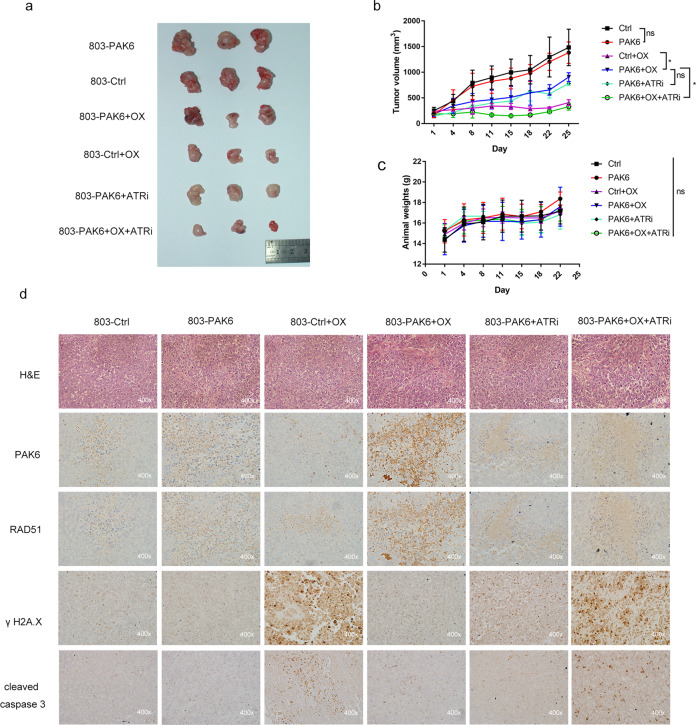
ATR inhibitor AZD6738 enhances antitumor efficacy of oxaliplatin in PAK6 overexpression cell line xenograft. **a** Tumor picture for each group was displayed. **b** Tumor growth of indicated 803 xenografts in each group. **c** Animal weight for indicated treatments. **d** HE staining and IHC staining of PAK6, RAD51, γH2A.X, and cleaved caspase 3 for the tumor isolated from the indicated treatments. Data presented as mean ± SD of three independent replicates. OX: oxaliplatin; ATRi: ATR inhibitor AZD6738; *: *P* < 0.05, ns: no statistical difference.

Considering the effectiveness of a combined oxaliplatin and AZD6738 therapy observed in vitro, the current study hypothesized whether the antitumor efficacy was also extended to in vivo models. The tumor volumes of the PAK6 overexpression xenografts were indistinguishable from that of control cells in the untreated groups (Fig. 7a, b). However, a single-agent treatment of oxaliplatin showed low growth inhibition in the PAK6 overexpression xenografts as compared with the control group and hence, indicated a chemoresistance of PAK6 in vivo. On the other hand, in the treatment of combination of oxaliplatin and AZD6738, the tumor volumes of the PAK6 overexpression xenografts were significantly reduced as compared with the volumes of the monotherapy of oxaliplatin (Fig. 7a, b). Moreover, the weights of the mice showed no significant difference among different treatment groups, indicating the drugs were tolerated (Fig. 7c).

The present study also treated the PAK6 overexpression xenografts with AZD6738 alone to assess the impact of AZD6738 on tumor growth, however, no significant difference was found as compared with the oxaliplatin monotherapy (Fig. 7a, b). Moreover, the drug synergy of oxaliplatin and AZD6738 on tumors were evaluated by measuring RAD51, γH2A.X, and cleaved-caspase-3 after tumors harvest. It was evident that γH2AX and cleaved caspase 3 was increased after combination of oxaliplatin and AZD6738 as compared with that in the monotherapy (Fig. 7d). Taken together, utility of the combination of oxaliplatin and AZD6738 in vivo experiment recapitulated the in vitro findings, indicating that this drug combination targets expected ATR pathway effectors in xenografts models.

## Discussion

Oxaliplatin is a third-generation platinum-based compound that has a favorable toxicity profile and ease of administration. It is hence commonly used as the first-line chemotherapy drug for the treatment of patients with GC [4, 21, 22]. Further, oxaliplatin exerts its anticancer effects through formation of platinum-DNA adducts, thereby inducing DNA damage and consequently resulting in cell apoptosis [23]. However, the anticancer effects of oxaliplatin are decreased because of the chemoresistance during the chemotherapy. Combinational therapy with additional chemotherapeutic drugs, such as 5-FU, has become one of the systemic treatment strategies for improving the patients survival rate [24]. Nevertheless, patients are also plagued with toxic side effects induced by combinational therapy and hence result in, an increase of the disease burden [25]. Therefore, there is urgent need for a stable biomarker to predict the chemoresistance to oxaliplatin, and identify the potential mechanism as well as establish the potential prevention and treatment targets for improving the antitumor efficacy of oxaliplatin in patients with GC.

Herein, the present study identified the potential roles of PAK6 in chemoresistance to oxaliplatin and in DNA damage response pathway in patients with GC. Clinicopathological data, along with chemotherapeutic treatment data from a total of 129 patients with GC in our hospital revealed that PAK6 resulted in deeper invasive, more lymph nodes metastasis, and higher rate of recurrence postoperatively. Results of further analysis showed that PAK6 is associate with chemoresistance to oxaliplatin but not 5-FU. It was evident that chemosensitivity was increased after PAK6 knockdown but strongly decreased after PAK6 overexpression. Functional experiments demonstrated PAK6 can facilitate HR repair during the treatment with oxaliplatin. Mechanistically, PAK6 acts as an upper novel HR repair protein that promotes efficient HR repair via activating ATR/CHK1 pathway, thereby further promotes RAD51 recruitments and opposes DNA double strand breaks induced by oxaliplatin. Moreover, our study further identified that ATR inhibitor AZD6738 can be used as a combinational drug with oxaliplatin to overcome the chemoresistance to oxaliplatin induced by PAK6 in vitro and in vivo, providing a potential possibility for future consideration in clinical cancer treatment.

In the recent years, PAK6 has been identified as oncogenic protein in different types of cancers like colorectal cancer [26], hepatocellular carcinoma [27], cervical cancer [28], prostate cancer [13], and gastric cancer [14]. Moreover, PAK6 has been found to be associated with radiosensitivity and chemosensitivity in prostate cancer [13, 29], chronic myeloid leukemia [12], and colon cancer [26], indicating the important role of PAK6 in cancer treatment response. However, these studies mainly focused on the role of PAK6 in BAD phosphorylation, which is a proapoptotic member of the Bcl-2 family, and none of them could provide valuable treatment suggestion to solve the chemoresistance or radiation resistance in clinical treatment. In this study, PAK6 was identified as an upper HR repair protein which activate ATR/CHK1 pathway and, thereby promote RAD51 recruitment to DNA damaged site to enhance HR repair in respond to DNA damage induced by oxaliplatin. Moreover, it was evident that ATR inhibitor AZD6738 combinate with oxaliplatin can overcome chemoresistance induced by PAK6 in vitro and in vivo. Since the AZD6738 is currently used as a monotherapy and in combination with chemotherapy and radiotherapy in many clinical trials and gives very good prognosis, it may be applicated as a valuable and powerful anticancer agent in the near future [30]. Hence, the combination of oxaliplatin and AZD6738 is a promising combination strategy for clinical treatment decision in GC.

**Fig. 8:**
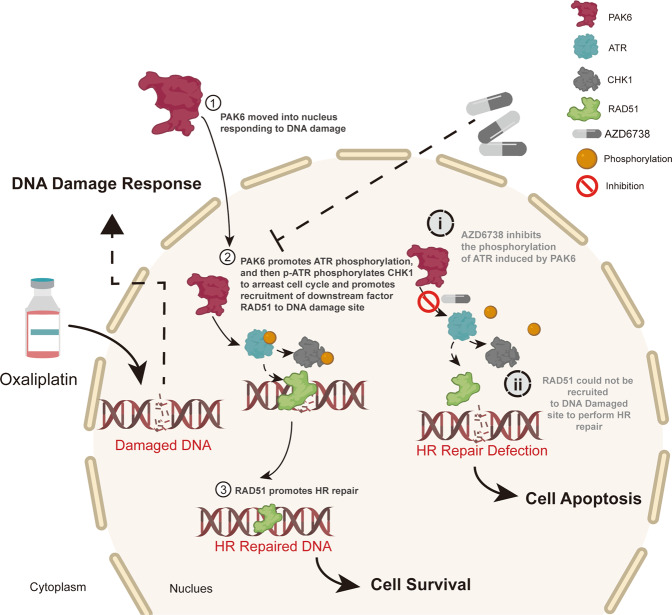
A model for PAK6 induced chemoresistance to oxaliplatin in GC. In PAK6 high expression GC cells, when DSB is initiated by the treatment of oxaliplatin, PAK6 could behave as an upper DDR factor to move into nucleus from cytoplasm to promote the phosphorylation of ATR. And then the activated p-ATR could further phosphorylate CHK1 to arrest the cell cycle in G2/M phase, and promote the recruitment of RAD51 to the nucleus to perform HR repair, thereby promoting the chemoresistance to oxaliplatin, ultimately leading to cells survival. However, failure to maintain the appropriate phosphorylation of ATR after conducting AZD6738 based on oxaliplatin treatment results in deficiency of recruitment of RAD51 to the nucleus to perform HR repair, ultimately leading to cell apoptosis. Combination of AZD6738 and oxaliplatin could overcome the chemoresistance to oxaliplatin induced by PAK6.

The main DNA repair pathways include predominant sensors, signaling, and effector proteins [31]. When severe DNA damage like DNA double strand breaks happens, the MRE11–RAD50–NBS1 complex (MRN) could act as the sensor to initiate to HR repair, and then ATR as well as ATM acts as the signaling proteins to activate the cascade proteins such as CHK1 to create the favorable conditions for HR repair, and also recruits effector protein such as RAD51 to perform the HR repair [32–34]. In this study, it was found that PAK6 could plays as an upper-stream protein in HR pathway to rapidly moves into nucleus in response to DSBs induced by oxaliplatin. The PAK6 then activates ATR/CHK1 signaling, and hence promote RAD51 to recruit into nucleus to enhance HR (Fig. 8). It was also found that even the ATR signaling was blocked, the RAD51 foci of nucleus was still increased in the PAK6 overexpression cells, indicating PAK6 may has the potential to carry RAD51 directly to the nucleus to enhance HR repair. Interestingly, PAK6 was found to interacts with RAD51 in GC cells in the exposure to oxaliplatin (Fig. S9a, b). Moreover, results of IF and immunohistochemical staining also demonstrated the co-localization of the PAK6 and RAD51, hence suggesting the interaction of PAK6 and RAD51. Previous studies have proved that RAD51, especially the nuclear part of RAD51, is the core protein in HR [35–37]. Therefore, any possibility that increases the nuclear recruitment of RAD51 may facilitate the HR efficacy. Considering the central role of RAD51 in HR, it is only logical that PAK6 promotes HR. Thus, PAK6 can on the one hand, moves into nucleus to activate ATR/CHK1 signaling promote HR repair, on the other hand, carries RAD51 into nucleus to further enhance HR repair. Nonetheless, substantial work lies ahead to fully determine the regulation of this RAD51 mediator in relation to HR. Moreover, although PAK6 was found to translocate into nucleus in response to treatment of oxaliplatin, the upper stream signaling to regulate the translocation of PKA6 remains unknow. Ramneet Kaur et al. reported that the activation of PAK6 could be inhibited by p38 mitogen-activated protein (MAP) kinase inhibitor, indicating that PAK6 plays a role in the cellular response to stress-related signals [38]. Since oxaliplatin could initiate the cellular stress to induce cell apoptosis, the translocation of PAK6 into nucleus may be regulated by stress-related signals. However, additional studies are needed to confirm this hypothesis.

In conclusion, it is evident that the PAK6 is not only a stable biomarker to identify the chemoresistance to oxaliplatin in GC, but is also an important HR repair factor that demonstrated a novel function in activating ATR/CHK1 signaling to regulate HR repair. Moreover, it was noted that the combination of ATR inhibitor AZD6738 and oxaliplatin are a potential treatment strategy for reversal of the chemoresistance to oxaliplatin induced by PAK6 in patients with GC.

## Materials and methods

### Patients and specimens

A total of 129 paraffin-embedded samples were obtained from patients with GC undergoing surgery at Southern Medical University Nanfang Hospital from 2014-2015. All the patients were histologically diagnosed with GC. Data on the characteristics about gender, age, CEA, CA199, TNM stage, chemotherapy treatment, and follow-up were collected for further analysis. The Cancer Genome Atlas Program (TCGA) dataset regarding gastric cancer (STAD, Stomach adenocarcinoma) was also downloaded and the current study was approved by the Nanfang Hospital Ethics Review Board.

### Immunohistochemistry staining and scoring

Consistent with the description of previous studies, Formalin-fixed paraffin-embedded samples were processed for immunohistochemistry (IHC) staining [14, 39]. Detailed information on the IHC staining was provided in supplementary materials. The H-score was used to quantify the expression of PAK6. The staining intensity was evaluated as negative (scored as 0), weak (scored as 1), intermediate (scored as 2), or strong (scored as 3) by two independent experienced pathologists.

The H-score (between 0 and 300) for each sample was calculated in the following way:

(% of cells stained at intensity 1 × 1) + (% of cells stained at intensity 2 × 2) + (% of cells stained at intensity 3 × 3).

The median was used as the cut-off value to classify the PAK6 expression into High- and Low-expressions.

### Cell culture

The HEK 293 T cells and human gastric cancer lines (AGS, BGC-823, HGC-27, MGC-803, and SGC-7901) were purchased from the Cell bank of Chinese Academy of Sciences (Shanghai, China). The HEK 293 T cells were cultured in DMEM (BI, Israel) supplemented with 10% FBS (BI, Israel) at 37 °C under 5% CO_2_.The AGS, BGC-823, HGC-27, MGC-803, and SGC-7901 cells were cultured in RPMI-1640 medium (BI, Israel) supplemented with 10% FBS at 37 °C under 5% CO_2_. All the cell lines were validated by mycoplasma testing and short tandem repeat (STR) profiling analyses.

### Transfections and infections

The PAK6 shRNA were packaged using HEK 293 T cells by Lenti-Pac HIV Expression Packaging Kit (GeneCopoeia) and then infected into AGS and BGC-823 cells. The PAK6, CS-HR-EGFP-M68, and CS-SceI-M23 expression constructs were transfected into HEK 293 T cells using Lipofectamine 3000 (Thermo Fisher Scientific, Waltham, MA).

Further, the lentivirus expressing PAK6 were infected into MGC-803 and SGC-7901 cells. Stable cell lines were selected with the presence of 500 ug/ml G418 (Biyuntian, China), 100 ug/ml hygromycin (Biyuntian, China), or 4 ug/ml puromycin (Biyuntian, China) according the relative resistance genes of constructs. Details about the products are showed in the supplementary materials.

### Quantitative real-time PCR analysis

Total RNA was extracted using an RNA-Quick purification kit (ES Science, Shanghai, China) and the cDNA synthesis was performed with PrimeScript RT Master Mix (RR036A, Takara, Japan) per the manufacturers’ instructions. Quantitative PCR analysis was performed on a QuantStudio 5 Ssytem with TaKaRa SYBR® Premix Taq™ (TbI RNase H Plus; TaKaRa, RR420). In the present study, all samples were assayed in triplicate. Relative expression levels were determined with the ΔΔCt method and normalized to the mean of housekeeping genes GAPDH. The primers utilized in this study were as showed in supplementary materials.

### Western Blot (WB) and Co-Immunoprecipitation (Co-IP)

Total proteins extracted using RIPA buffer with 1 mmol/L phenylmethylsulfonyl fluoride (PMSF) and protease inhibitor cocktail (Roche, Germany) were normalized using BCA protein kit and degenerated using sodium dodecyl sulfate polyacrylamide gel electrophoresis (SDS-PAGE) loading buffer. Total proteins were separated with 8-12% SDS-PAGE and transferred to polyvinylidene fluoride (PVDF) membranes. Subsequently, the membranes were blocked and incubated overnight with a primary antibody at 4 °C. The ECL enhanced chemiluminescence substrate kit (Millipore) was used for imaging and quantitation after incubation with secondary antibodies using the Image J software.

Cytoplasmtic and nuclear proteins were extracted using nuclear and cytoplasmtic extraction reagents (BestBio) according the manufacturer’s instructions. The Co-IP of the lysate was performed using the Magnetic Co-Immunoprecipitation (Co-IP) Kit (Biosense) according to the protocol provided by the manufacturer. Further, the obtained supernatants were divided into three parts: 10% for using as input protein; 40% for incubating with IgG antibody, and 40% for incubation with specific primary antibodies (PAK6, RAD51, p-ATR, and p-CHK1) at 4 °C overnight. The immunoprecipitates were analyzed using WB after being dissociated using magnetic beads and degenerated in loading buffer. The reagents and antibodies used in the present study are as listed in the supplementary materials.

### Cell viability and apoptosis assay

To study cell viability, the cells were plated at a density of 3000 cells per well in 96-well plates and treated with oxaliplatin (0, 2, 4, 8, 16, and 32 ug/ml) or 5-FU (0, 2, 4, 8, 16, 32, and 64 μg/ml) for 24 h. Cell counting kit (Dojindo Laboratories) was used to detected cell viability. For cell apoptosis assay, cells were plated at a density 1 × 10^5^ cells per well in 6-well plates and treated with oxaliplatin (8 ug/ml) for 2 days. The apoptotic rate was determined with Annexin V-FITC/APC (BD Biosciences) and DAPI using FACSCalibur flow cytometer (BD Biosciences, USA) and analyzed using FlowJo software (TreeStar, OR, USA).

### Clonogenic survival assays

For Clonogenic survival assays, the cells were plated at a density of 500 cells in 6-well plates in technical triplicate. For drug sensitivity of PAK6 overexpression cells, oxaliplatin was added in a low concentration (0.5 ug/ml) 24 h following plating and cells were incubated for a further 10 days. For drug sensitivity of PAK6 knockdown cells, oxaliplatin was added in a low concentration (0.3 μg/ml) 24 h following plating and cells were incubated for a further 12 days. The cells treated with oxaliplatin were normalized to untreated cells.

### Immunofluorescence

Cells were plated at the confocal microscope plates (NEST) and incubated with oxaliplatin (8 μg/ml) at indicated time points. After washed by PBS for 3 times, cells were fixed with 4% paraformaldehyde for 15 min at room temperature and then permeabilized with 0.1% Triton X-100 for 15 min at room temperature. Being blocked with 5% BSA in PBST for 1 h at room temperature, cells were incubated overnight with PAK6, γH2A.X, or RAD51 antibodies at 4 °C. Thereafter, the cells were incubated with Alexa Fluor 488-labeled Goat Anti-Mouse IgG and Alexa Fluor 647-labeled Goat Anti-Rabbit IgG for 1 h at room temperature. Nucleus were counterstained with DAPI. Then images were acquired by a laser scanning confocal microscope LSM980 (Zeiss).

### Comet assay

The presence of DNA damage was analyzed using the Comet Assay kit (4250-050-K, Trevigen, Gaithersburg, MD) according to the manufacturer’s instructions. Cells were combined with Comet Agarose and spread onto Comet Assay slides, and then were electrophoresed in neutral solution followed by staining with SYBR Green or PI (Invitrogen). Images were captured using an inverted microscope (Olympus). At least 30 cells were analyzed per sample using CASP software. The DNA damage was measured by the tail moment.

### Homologous recombination (HR) repair

The efficiency of HR repair was assessed using an I-SceI expression plasmid (CS-SceI-M23) and an I-SceI repair reporter plasmid (CS-HR-EGFP-M68) composed of mutated EGFP that contained a unique I-SceI restriction site. The gene conversion repair of the double-strand break (DSB) that was caused by I-SceI restriction could reflect the efficiency of HR repair. In brief, the HEK 293 T cells were first transfected with 5 ug of HR-EGFP plasmid to construct the HR reporter cells (293T-HR-EGFP cells). The 293T-HR-EGFP cells were then transfected with PAK6 expression or empty control plasmids. After screening out the stably transfected cells, the myc-targeted I-SceI constructs were transfected into the 293T-HR-EGFP-PAK6 cells and 293T-HR-EGFP-Vec cells. At 24 h, after I-SceI transfection, the cells were harvested and the number of EGFP positive cells was detected using FACS Calibur flow cytometer (BD Biosciences, USA). Protein of the reporter cells was further analyzed using western blotting.

### Inhibitor implementation

The ATR inhibitor (AZD6738) and ATM inhibitor (AZD0156) were used as the combined agents with oxaliplatin in vitro or in vivo. To measure the cytotoxicity of the inhibitors, the cells were seeded in triplicates in 96-well plates and incubated with different concentrations of inhibitors (0, 0.01, 0.1, 0.2, 0.3, and 0.4 μM) for 48 h followed by the cell viability test with CCK8 reagent. The maximum concentration that showed complete absence of cytopathic effect in an individual culture well was defined as the best concentration. For in vitro experiments, ATR inhibitor and ATM inhibitor were implemented with the best concentration. For in vivo experiments, ATR inhibitor was used at a recommend concentration of 25 mg/kg/mouse/day [40].

### In vivo tumor experiments

All animal experiments were carried out with the consent of the Ethics Committee of Southern Medical University Nanfang Hospital. The PAK6 overexpression MGC-803 cells or empty control MGC-803 cells were subcutaneously administered into the left hind limb of 6-week-old female BALB/c nude mice. Details were showed in the supplementary materials.

### Gene set enrichment analysis

GSEA was performed using the GSEA version 3.0 to identify related biological processes, based on the TCGA dataset regarding gastric cancer (STAD, Stomach adenocarcinoma). A null distribution for enrichment score (ES) was generated using 1000 gene permutations. Then, a normalization enrichment score (NES) was calculated for each enriched pathway. The false discovery rate (FDR) < 5% and the *P* value < 0.05 were considered to be statistically significant.

### Statistical analysis

Statistical analysis was performed using SPSS version 22.0 and GraphPad Prism version 8 software. Correlations between the expression levels of PAK6 and clinical characteristics were assessed using Spearman correlation analyses. Kaplan–Meier curves and log-rank test were used to compare the prognosis between the patients with high expression of PAK6 and those with low expression of PAK6. Quantitative data are expressed as mean ± SD. The two-tailed Student *t* test or one-way analysis of variance were used to determine the statistical significance. Each experiment was replicated at least thrice. The *P* value < 0.05 was considered as statistically significant.

## Supplementary information

Supplementary materials

aj-checklist

Original full length western blots

## Data Availability

All data used in this study are available from the corresponding authors on reasonable request.

## References

[1] Smyth EC, Nilsson M, Grabsch HI, van Grieken NCT, Lordick F (2020). Gastric cancer. Lancet.

[2] Sung H, Ferlay J, Siegel RL, Laversanne M, Soerjomataram I, Jemal A (2021). Global Cancer Statistics 2020: GLOBOCAN Estimates of Incidence and Mortality Worldwide for 36 Cancers in 185 Countries. CA Cancer J Clin.

[3] Thrift AP, El-Serag HB (2020). Burden of gastric cancer. Clin Gastroenterol Hepatol.

[4] Al-Batran S-E, Hartmann JT, Probst S, Schmalenberg H, Hollerbach S, Hofheinz R (2008). Phase III trial in metastatic gastroesophageal adenocarcinoma with fluorouracil, leucovorin plus either oxaliplatin or cisplatin: a study of the Arbeitsgemeinschaft Internistische Onkologie. J Clin Oncol.

[5] Raymond E, Chaney SG, Taamma A, Cvitkovic E (1998). Oxaliplatin: a review of preclinical and clinical studies. Ann Oncol.

[6] Charalampakis N, Economopoulou P, Kotsantis I, Tolia M, Schizas D, Liakakos T (2018). Medical management of gastric cancer: a 2017 update. Cancer Med.

[7] Rane CK, Minden A (2019). P21 activated kinase signaling in cancer. Semin Cancer Biol.

[8] Radu M, Semenova G, Kosoff R, Chernoff J (2014). PAK signalling during the development and progression of cancer. Nat Rev Cancer.

[9] Babagana M, Johnson S, Slabodkin H, Bshara W, Morrison C, Kandel ES (2017). P21-activated kinase 1 regulates resistance to BRAF inhibition in human cancer cells. Mol Carcinog.

[10] Zheng J, Zhang C, Li Y, Jiang Y, Xing B, Du X (2021). p21-activated kinase 6 controls mitosis and hepatocellular carcinoma progression by regulating Eg5. Biochim Biophys Acta Mol Cell Res.

[11] Li T, Li Y, Liu T, Hu B, Li J, Liu C (2020). Mitochondrial PAK6 inhibits prostate cancer cell apoptosis via the PAK6-SIRT4-ANT2 complex. Theranostics.

[12] Lin H, Rothe K, Chen M, Wu A, Babaian A, Yen R (2020). The miR-185/PAK6 axis predicts therapy response and regulates survival of drug-resistant leukemic stem cells in CML. Blood.

[13] Wen X, Li X, Liao B, Liu Y, Wu J, Yuan X (2009). Knockdown of p21-activated Kinase 6 Inhibits Prostate Cancer Growth and Enhances Chemosensitivity to Docetaxel. Urology.

[14] Jiang Y, Liu W, Li T, Hu Y, Chen S, Xi S (2017). Prognostic and Predictive Value of p21-activated Kinase 6 Associated Support Vector Machine Classifier in Gastric Cancer Treated by 5-fluorouracil/Oxaliplatin Chemotherapy. EBioMedicine.

[15] Vancevska A, Douglass KM, Pfeiffer V, Manley S, Lingner J (2017). The telomeric DNA damage response occurs in the absence of chromatin decompaction. Genes Dev.

[16] Bothmer A, Robbiani DF, Di Virgilio M, Bunting SF, Klein IA, Feldhahn N (2011). Regulation of DNA end joining, resection, and immunoglobulin class switch recombination by 53BP1. Mol Cell.

[17] Zhao W, Steinfeld JB, Liang F, Chen X, Maranon DG, Jian Ma C (2017). BRCA1-BARD1 promotes RAD51-mediated homologous DNA pairing. Nature.

[18] Motegi A, Masutani M, Yoshioka K-I, Bessho T (2019). Aberrations in DNA repair pathways in cancer and therapeutic significances. Semin Cancer Biol.

[19] Bradbury A, Hall S, Curtin N, Drew Y (2020). Targeting ATR as Cancer Therapy: A new era for synthetic lethality and synergistic combinations?. Pharm Ther.

[20] Colomer C, Margalef P, Villanueva A, Vert A, Pecharroman I, Solé L (2019). IKKα kinase regulates the DNA damage response and drives chemo-resistance in cancer. Mol Cell.

[21] Yagi S, Yamada K, Terayama M, Wake H, Enomoto N, Nohara K (2021). Current status of doublet combinations of platinum and fluoropyrimidines using oxaliplatin for advanced gastric cancer. Glob Health Med.

[22] Cunningham D, Starling N, Rao S, Iveson T, Nicolson M, Coxon F (2008). Capecitabine and oxaliplatin for advanced esophagogastric cancer. N Engl J Med.

[23] Faivre S, Chan D, Salinas R, Woynarowska B, Woynarowski JM (2003). DNA strand breaks and apoptosis induced by oxaliplatin in cancer cells. Biochem Pharm.

[24] Yamauchi K, Yang M, Hayashi K, Jiang P, Yamamoto N, Tsuchiya H (2008). Induction of cancer metastasis by cyclophosphamide pretreatment of host mice: an opposite effect of chemotherapy. Cancer Res.

[25] Hammann F, Gotta V, Conen K, Medinger M, Cesana P, Rochlitz C (2017). Pharmacokinetic interaction between taxanes and amiodarone leading to severe toxicity. Br J Clin Pharm.

[26] Chen J, Lu H, Yan D, Cui F, Wang X, Yu F (2015). PAK6 increase chemoresistance and is a prognostic marker for stage II and III colon cancer patients undergoing 5-FU based chemotherapy. Oncotarget.

[27] Chen H, Miao J, Li H, Wang C, Li J, Zhu Y (2014). Expression and prognostic significance of p21-activated kinase 6 in hepatocellular carcinoma. J Surg Res.

[28] Yang Q, Zhao Y, Chen Y, Chang Y, Huang A, Xu T (2020). PAK6 promotes cervical cancer progression through activation of the Wnt/β-catenin signaling pathway. Oncol Lett.

[29] Zhang M, Siedow M, Saia G, Chakravarti A (2010). Inhibition of p21-activated kinase 6 (PAK6) increases radiosensitivity of prostate cancer cells. Prostate.

[30] Foote KM, Nissink JWM, McGuire T, Turner P, Guichard S, Yates JWT (2018). Discovery and characterization of AZD6738, a potent inhibitor of ataxia telangiectasia mutated and Rad3 related (ATR) kinase with application as an anticancer agent. J Med Chem.

[31] Brown JS, Carrigan B, Jackson SP, Yap TA (2017). Targeting DNA repair in cancer: beyond PARP inhibitors. Cancer Discov.

[32] Perkhofer L, Gout J, Roger E, Kude de Almeida F, Baptista Simoes C, Wiesmuller L (2021). DNA damage repair as a target in pancreatic cancer: state-of-the-art and future perspectives. Gut.

[33] Sørensen CS, Hansen LT, Dziegielewski J, Syljuåsen RG, Lundin C, Bartek J (2005). The cell-cycle checkpoint kinase Chk1 is required for mammalian homologous recombination repair. Nat Cell Biol.

[34] Sun Y, McCorvie TJ, Yates LA, Zhang X (2020). Structural basis of homologous recombination. Cell Mol life Sci: CMLS.

[35] Laurini E, Marson D, Fermeglia A, Aulic S, Fermeglia M, Pricl S (2020). Role of Rad51 and DNA repair in cancer: a molecular perspective. Pharm Ther.

[36] Godin SK, Sullivan MR, Bernstein KA (2016). Novel insights into RAD51 activity and regulation during homologous recombination and DNA replication. Biochem Cell Biol.

[37] Krejci L, Altmannova V, Spirek M, Zhao X (2012). Homologous recombination and its regulation. Nucleic Acids Res.

[38] Kaur R, Liu X, Gjoerup O, Zhang A, Yuan X, Balk SP (2005). Activation of p21-activated Kinase 6 by MAP Kinase Kinase 6 and p38 MAP Kinase *. J Biol Chem.

[39] Jiang Y, Zhang Q, Hu Y, Li T, Yu J, Zhao L (2018). ImmunoScore signature: a prognostic and predictive tool in gastric cancer. Ann Surg.

[40] Lloyd RL, Wijnhoven PWG, Ramos-Montoya A, Wilson Z, Illuzzi G, Falenta K (2020). Combined PARP and ATR inhibition potentiates genome instability and cell death in ATM-deficient cancer cells. Oncogene.

