# Supplementary material for: PAK6 promotes homologous-recombination to enhance chemoresistance to oxaliplatin through ATR/CHK1 signaling in gastric cancer
Source: Cell Death Dis. 2022 Jul 28;13(7):658. doi: 10.1038/s41419-022-05118-8 (PMC9334622; doi:10.1038/s41419-022-05118-8)
Supplement: Supplementary file 1 — Supplementary materials [file 41419_2022_5118_MOESM1_ESM.docx]

**Supporting information**

**Supplemental Experimental Procedures**

**Immunohistochemistry staining and scoring**

In this study, PAK6 was stained. Formalin-fixed paraffin-embedded (FFPE) samples were processed for IHC staining as previously described^1, 2^. The samples were incubated with rabbit polyclonal to PAK6 (ab154752) antibody. Every staining run contained a slide treated with phosphate buffer saline (PBS) buffer in place of the primary antibody as a negative control. Every staining run contained a slide of positive control. Prior to staining, sections were blocked with endogenous peroxidase (prepared in 1% H_2_O_2_/methanol solution) for 10 minutes and then microwaved for 30 minutes in 10 mM citrate buffer, pH 6.0. The sections were blocked using 10% normal goat serum for 30 minutes. Furthermore, all slides were stained with the same concentrations of primary antibody overnight at 4 ℃, followed by incubation with an amplification system with a labeled polymer/HRP (EnVision™, DakoCytomation, Denmark) at 37℃ for 30 minutes. The sections were developed with 0.05% 3, 3´-diaminobenzidine tetrahydrochloride (DAB) and counterstained with modified Harris hematoxylin. And all slides were stained with DAB dyeing for the same time for each antibody. Two pathologists (T.L. and S.X. with 5 to 10 years of experience) who were blinded to clinical outcomes independently scored all samples. A third pathologist was consulted when a difference of opinion arose between the 2 primary pathologists. At low power (100), the tissue sections were screened using an inverted research microscope (model DM IRB; Leica, Germeny), and the 4 most representative fields were selected. Thereafter, to evaluate the density of PAK6, the respective area of tumor was measured at 200 magnification.

**Quantitative Real-Time PCR Analysis**

The primers utilized in this study were listed as follows:

| **primers** | **5’ to 3’** |
| --- | --- |
| PAK6-For | GCTCTCGGACTTCGGATTCT |
| PAK6-Rev | GGCATACAAAGACCTGGAGAT |
| GAPDH-For | ACGGATTTGGTCGTATTGGG |
| GAPDH-Rev | CGCTCCTGGAAGATGGTGAT |
| ATR-For | GGCCAAAGGCAGTTGTATTGA |
| ATR-Rev | GTGAGTACCCCAAAAATAGCAGG |
| ATM-For | ATCTGCTGCCGTCAACTAGAA |
| ATM-Rev | GATCTCGAATCAGGCGCTTAAA |
| CHK1-For | ATATGAAGCGTGCCGTAGACT |
| CHK1-Rev | TGCCTATGTCTGGCTCTATTCTG |
| RAD51-For | CAACCCATTTCACGGTTAGAGC |
| RAD51-Rev | TTCTTTGGCGCATAGGCAACA |

**shRNA, plasmids, lentivirus, reagents and antibodies**

PAK6 shRNA (Catalog#: HSH107343-LVRU6GP-a and HSH107343-LVRU6GP-c) and the corresponding Scrambled shRNA control (Catalog#: CSHCTR001-LVRU6GP) constructs with green fluorescent protein (GFP) and puromycin resistance gene were obtained from GeneCopoeia. PAK6 (Catalog#: EX-W1277-M09), CS-HR-EGFP-M68 (Catalog#: CS-HR-EGFP-M68) and CS-SceI-M23 (Catalog#: CS-SceI-M23) expression constructs were purchased from Genecopoeia. Lentivirus expressing Cherry/PAK6 (LV-PAK6) was constructed by Genechem (Shanghai, China) using Ubi-MCS-3FLAG-SV40-Cherry-IRES-puromycin ctrltor and Ubi-MCS-3FLAG-SV40-Cherry-IRES-puromycin empty ctrltors were used as controls (Shanghai Genechem Co. Ltd., China). Oxaliplatin (HY-17371), 5-Fluorouracil (HY-90006), Ceralasertib (AZD6738), AZD0156 (HY-100016) were purchased from MedChemExpress. Rabbit polyclonal to PAK6 (ab154752) antibody was purchased from Abcam. Mouse monoclonal PAK6 antibody (sc-393075), Mouse monoclonal ATM (ATM 11G12), and Mouse monoclonal Phospho-Histone H2A.X (Ser139) Rad51 (sc-398587) were purchased from Santa Cruz. Rabbit polyclonal ATR, Rabbit polyclonal to CHK2 (bs-1391R), Phospho-CHK2 (Ser28) antibody was purchased from Bioss. Phospho-ATR (Ser428), Phospho-ATM (Ser1981) mouse mAb, CHK1 (2G1D5) mouse mAb, Phospho-CHK1 (Ser345) rabbit mAb, 53BP1, Phospho-53BP1 (Ser1618), Histone H2A.X (D17A3) rabbit mAb, Phospho-Histone H2A.X (Ser139), Cleaved Caspase 3 (9661) were purchased from Cell Signaling Technology; Mouse monoclonal to GAPDH (60004-1-lg), HRP-conjugated Affinipure Goat Anti-Mouse IgG(H+L) (SA00001-1), HRP-conjugated Affinipure Goat Anti-Rabbit IgG(H+L) (SA00001-2) were purchased from Proteintech. Alexa Fluor 488-labeled Goat Anti-Mouse IgG(H+L) (A0428), Alexa Fluor 647-labeled Goat Anti-Rabbit IgG(H+L) (A0468), and DAPI (C1005) were purchased from Beyotime.

***In vivo* tumor experiments**

Mice were randomly classified into six groups (N=3 per group) when the average tumor volume reached 100-150 mm^3^. In this study, two groups were injected with control cells, four groups were injected with PAK6 overexpression cells. In the control groups, one group was treated with oxaliplatin, and the other group was untreated; in the PAK6 groups, one group was treated with oxaliplatin, one group was untreated, one group was treated with AZD6738 alone, and the last group was treated with the combination of oxaliplatin and AZD6738. Oxaliplatin was dissolved in 5% glucose/H_2_O and intraperitoneally injected at a dose of 5 mg/kg/week in the oxaliplatin treated groups, and the absence of oxaliplatin in 5% glucose/H_2_O was injected at a dose of 5mg/kg/week as empty ctrltor in the AZD6738 alone group. AZD6738 was formulated in 10% DMSO/40% Propylene Glycol/50% H_2_O and orally dosed (25 mg/kg, 3days on and 4days off). In all combinations of AZD6738 was dosed 1 h after oxaliplatin injection. The width (W) and length (L) of the tumors were obtained with digital calipers, and the tumor volume was computed by the following formula: Volume (mm3) = (L x W^2^)/2. Mice body weight and tumor condition was recorded twice weekly for the duration of the treatment.

**Supplementary Table**

Table S1. Characteristics of patients with GC.

| **Variables** | **PAK6 expression** | | *P* |
| --- | --- | --- | --- |
|  | Low (N=64) % | High (N=65) % |  |
| **Age (years)** |  | | 0.926 |
| ≥60 | 34 (53.1) | 34 (52.3) |  |
| < 60 | 30 (46.9) | 31 (47.7) |  |
| **Gender** |  |  |  |
| Male | 46 (71.9) | 46 (70.8) | 0.89 |
| Female | 18 (28.1) | 19 (29.2) |  |
| **Size** |  |  | 0.429 |
| ≥4cm | 36 (56.3) | 41 (63.1) |  |
| < 4cm | 28 (43.7) | 24 (36.9) |  |
| **Differentiation** |  |  | 0.716 |
| Well | 2 (3.1) | 4 (6.2) |  |
| Moderate | 14 (21.9) | 14 (21.5) |  |
| Poor or undifferentiation | 48 (75.0) | 47 (72.3) |  |
| **Location** |  |  | 0.687 |
| Cardia | 4 (6.3) | 7 (10.8) |  |
| Body | 23 (35.9) | 25 (38.5) |  |
| Antrum | 35 (54.7) | 30 (46.1) |  |
| Whole | 2 (3.1) | 3 (4.6) |  |
| **CEA** |  |  | 0.35 |
| elevated | 8 (12.5) | 12 (18.5) |  |
| normal | 56 (87.5) | 53 (81.5) |  |
| **CA19-9** |  |  | 0.668 |
| elevated | 10 (15.6) | 12 (18.5) |  |
| normal | 54 (84.4) | 53 (81.5) |  |
| **CA724** |  |  | 0.129 |
| elevated | 9 (14.1) | 16 (24.6) |  |
| normal | 55 (85.9) | 49 (75.4) |  |
| **Lauren type** |  |  | 0.16 |
| intestinal | 21 (32.8) | 25 (38.4) |  |
| diffuse | 28 (43.8) | 33 (50.8) |  |
| mixed | 15 (23.4) | 7 (10.8) |  |
| **Borrmann type** |  |  | 0.13 |
| I | 3 (4.7) | 5 (7.7) |  |
| II | 5 (7.8) | 0 (0) |  |
| III | 49 (76.6) | 52 (80.0) |  |
| IV | 7 (10.9) | 8 (12.3) |  |
| **T stage** |  |  | 0.018* |
| T1 | 7 (10.9) | 3 (4.6) |  |
| T2 | 11 (17.2) | 4 (6.2) |  |
| T3 | 15 (23.4) | 13 (20.0) |  |
| T4a | 31 (48.5) | 39 (60.0) |  |
| T4b | 0 (0) | 6 (9.2) |  |
| **N stage** |  |  | 0.020* |
| N0 | 19 (29.7) | 10 (15.4) |  |
| N1 | 11 (17.2) | 4 (6.1) |  |
| N2 | 10 (15.6) | 12 (18.5) |  |
| N3 | 24 (37.5) | 39 (60.0) |  |
| **M stage** |  |  | 0.197 |
| M0 | 61 (95.3) | 58 (89.2) |  |
| M1 | 3 (4.7) | 7 (10.8) |  |
| **Recurrence** |  |  | 0.004* |
| negative | 56 (87.5) | 43 (66.2) |  |
| positive | 8 (12.5) | 22 (33.8) |  |
| **Chemotherapy** |  |  | 0.303 |
| none | 21 (32.8) | 16 (24.6) |  |
| Capecitabine | 8 (12.5) | 5 (7.7) |  |
| oxaliplatin and 5-FU | 35 (54.7) | 44 (67.7) |  |

**Supplementary Figure**

**
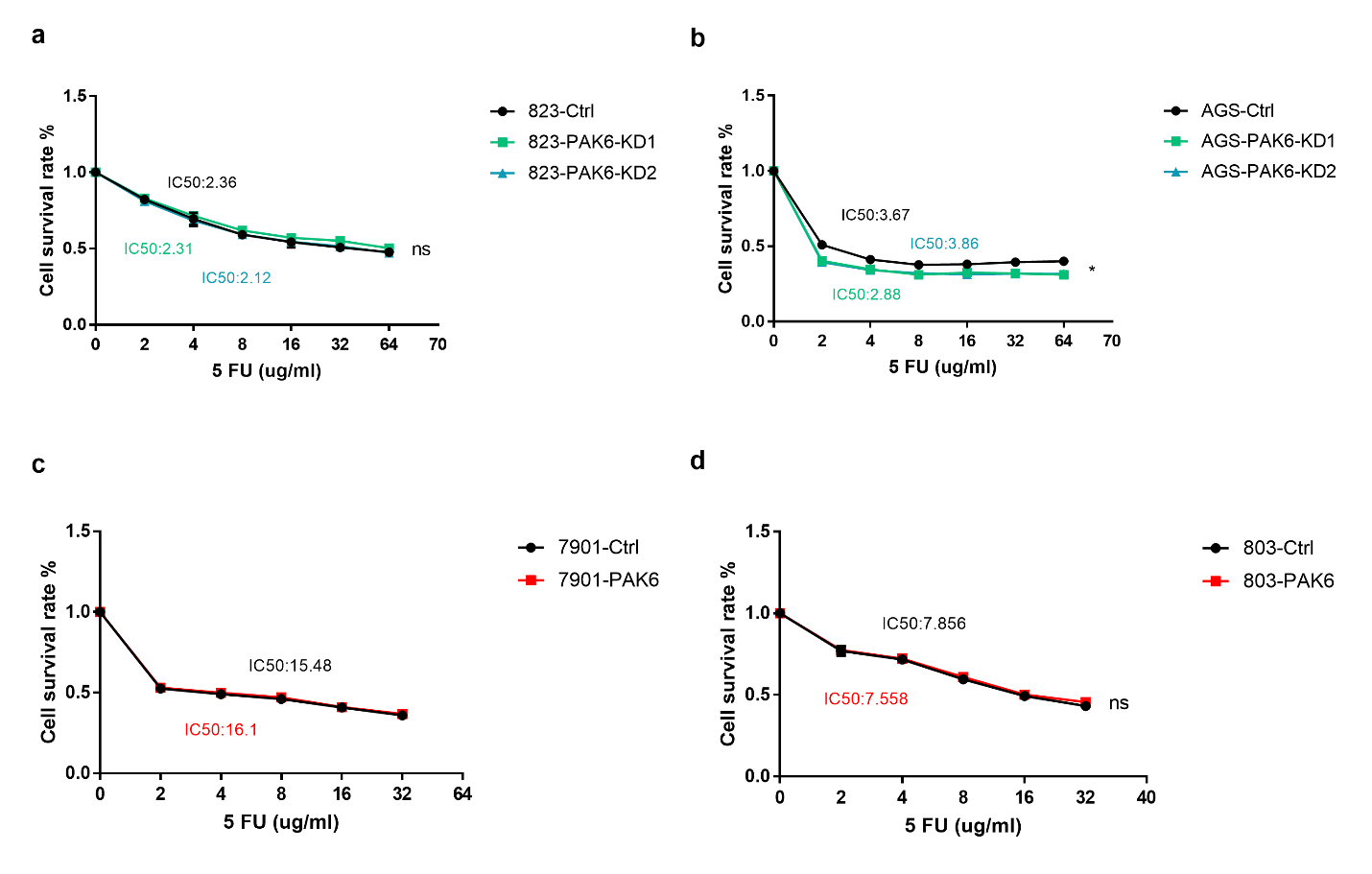
**

**Figure S1. PAK6 is not associated with the therapeutic efficacy of 5 FU in GC cells.** (a) Dose-response curves of control cells or PAK6 knocked down cells in 823 GC cell line after treated with 5 FU for 24 hours. The IC_50_ of the 5 FU in the cells are 2.36(823-Ctrl cells), 2.31 (823-PAK6-KD1 cells), and 2.12 (823-AK6-KD2 cells) μg/ml. (b) Dose-response curves of control cells or PAK6 knocked down cells in AGS GC cell line after treated with 5 FU for 24 hours. The IC_50_ of the oxaliplatin in the cells are 3.673 (AGS-Ctrl cells), 2.88 (AGS-PAK6-KD1 cells), and 3.86 (AGS-PAK6-KD2 cells) μg/ml. (c) Dose-response curves of control cells or PAK6 overexpression cells in 7901 GC cell line after treated with 5 FU for 24 hours. The IC_50_ of the oxaliplatin in the cells are 15.48 (7901-Ctrl cells) and 16.1 (7901-PAK6 cells) μg/ml. (n) Dose-response curves of control cells or PAK6 overexpression cells in 803 GC cell line after treated with 5 FU for 24 hours. The IC_50_ of the oxaliplatin in the cells are 7.856 (803-Ctrl cells) and 7.558 (803-PAK6 cells) μg/ml. Data presented as mean ± SD of three independent replicates. *: *P* < 0.05, ns: no statistical difference.


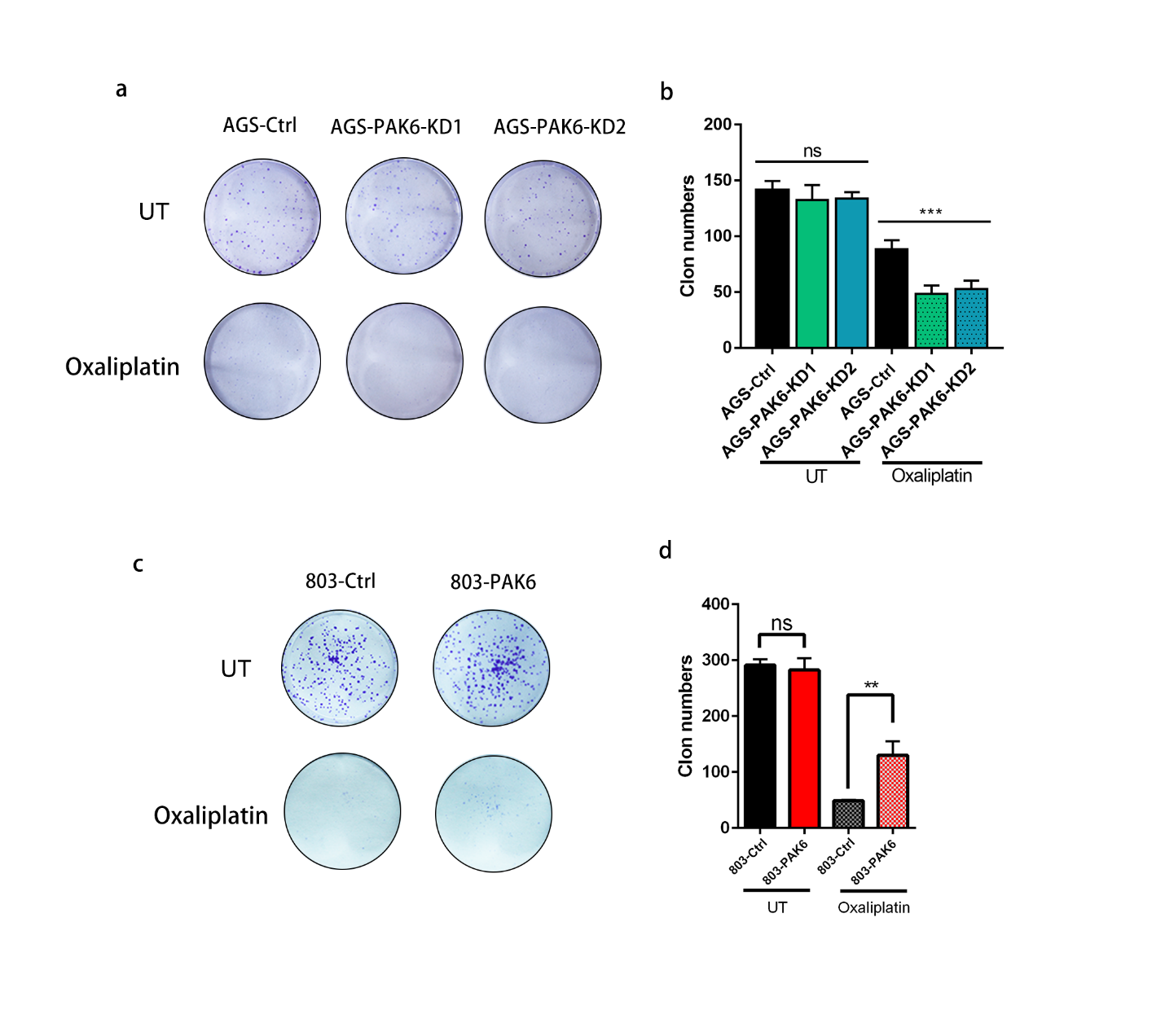


**Figure S2. PAK6 affects colony formation ability of GC cells in the presence of oxaliplatin.** (a) Colony formation ability of control cells and PAK6 knocked down cells in AGS GC cell line treated with or without oxaliplatin (0.3 μg/ml). (b) Clone numbers of control cells and PAK6 knocked down cells in AGS GC cell line treated with or without oxaliplatin. (c) Colony formation ability of control cells and PAK6 overexpression cells in 803 GC cell line treated with or without oxaliplatin (0.5 μg/ml). (d) Clone numbers of control cells and PAK6 overexpression cells in 803 GC cell line treated with or without oxaliplatin. Data presented as mean ± SD of three independent replicates. **: *P* < 0.01, ***: *P* < 0.001, ns: no statistical difference.


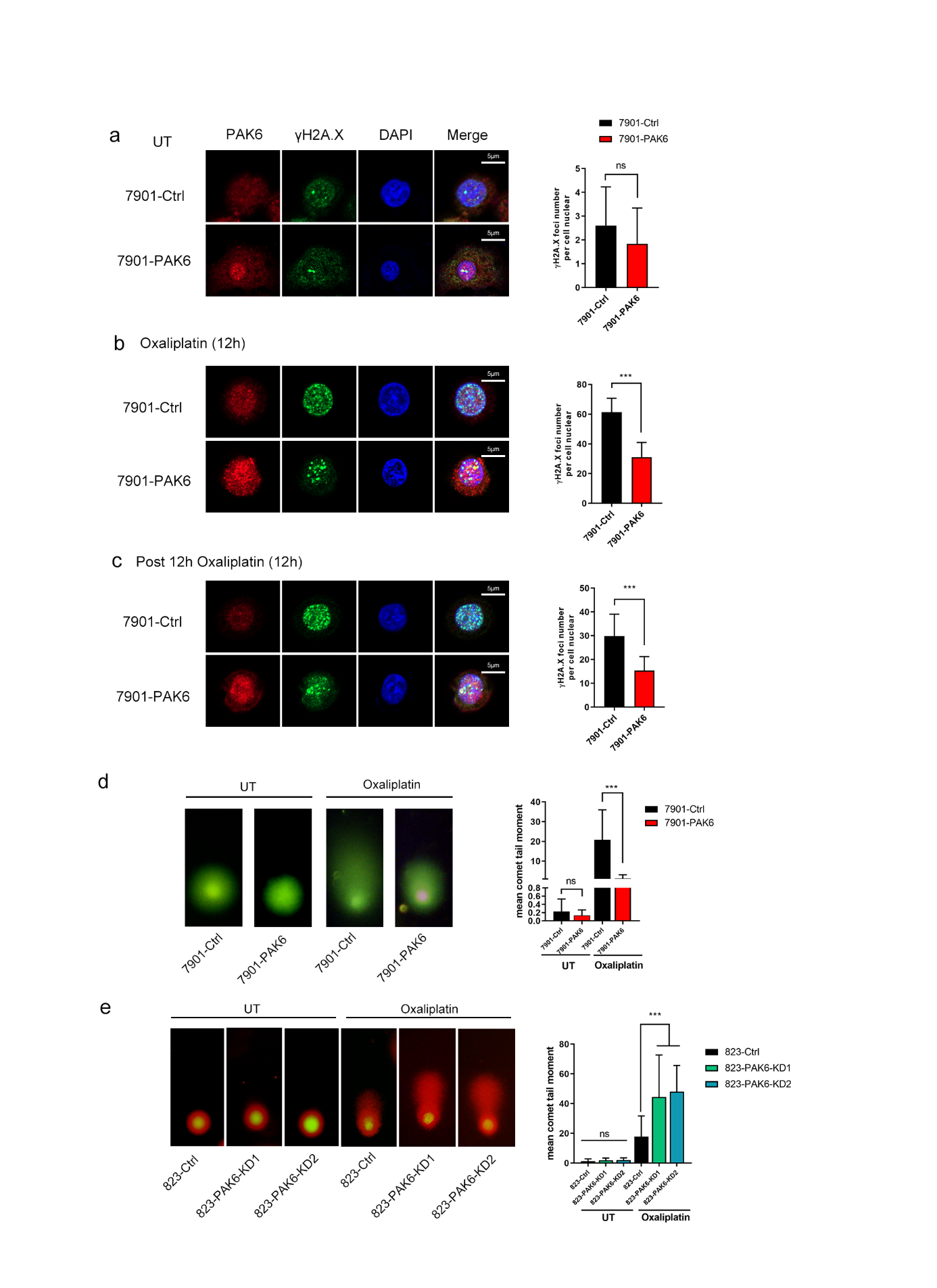


**Figure S3. PAK6 reduces the DNA damage induced by oxaliplatin.** (a) Left: PAK6 staining (red), γH2A.X staining (green), and DAPI staining (blue) in the control cells and PAK6 overexpression cells in 7901 GC cell line; Right: Quantification of mean γH2A.X foci per cell. (b) Left: PAK6 staining (red), γH2A.X staining (green), and DAPI staining (blue) in the control cells and PAK6 overexpression cells in 7901 GC cell line after treated with oxaliplatin for 12 hours; Right: Quantification of mean γH2A.X foci per cell. (c) Left: PAK6 staining (red), γH2A.X staining (green), and DAPI staining (blue) in the control cells and PAK6 overexpression cells in 7901 GC cell line after recovery from oxaliplatin treating (12 hours) for 12 hours; Right: Quantification of mean γH2A.X foci per cell. At least 30 cells per group were included for the counting and quantification. (d-e) Left: Representative images of neutral comet assays of control cells, PAK6 overexpression cells, and PAK6 knocked down cells in the absence and presence of oxaliplatin; Right: Quantification of the percentages of DNA tail moments. At least 30 cells per group were included for the counting and quantification. UT: untreated. ***: *P* < 0.001, ns: no statistical difference.


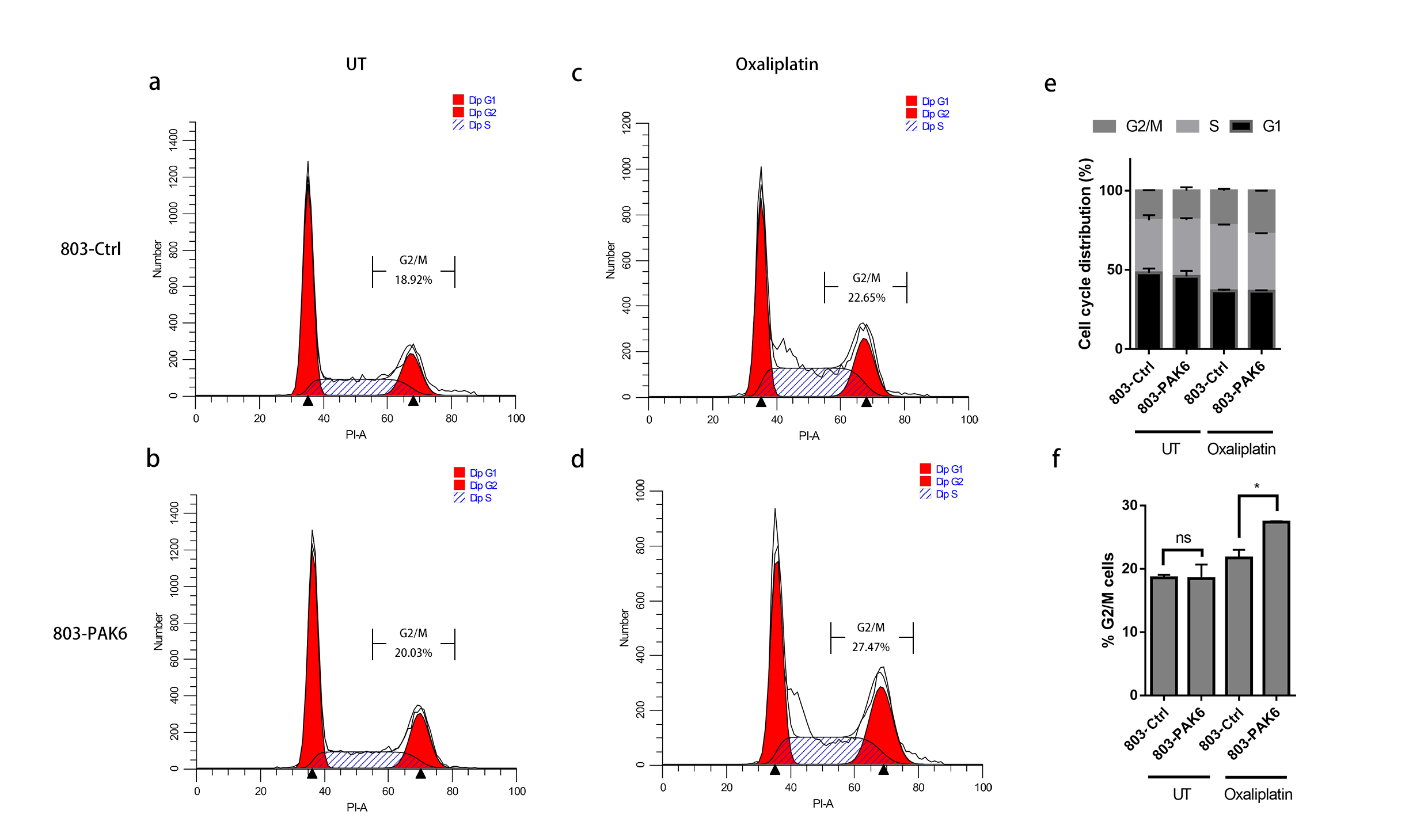


**Figure S4. PAK6 results in G2/M cell cycle arrest after treated with oxaliplatin.** (a) Cell cycle of control cells in 803 GC cell line. (b) Cell cycle of PAK6 overexpression cells in 803 GC cell line. (c) Cell cycle of control cells treated with oxaliplatin for 24 h in 803 GC cell line. (d) Cell cycle of PAK6 overexpression cells treated with oxaliplatin for 24 h in 803 GC cell line. (e) Cell cycle distribution of indicated groups. (f) Percentage of G2/M phase arrested cells of indicated groups. Data presented as mean ± SD of twice independent replicates. *: *P* < 0.05, ns: no statistical difference.


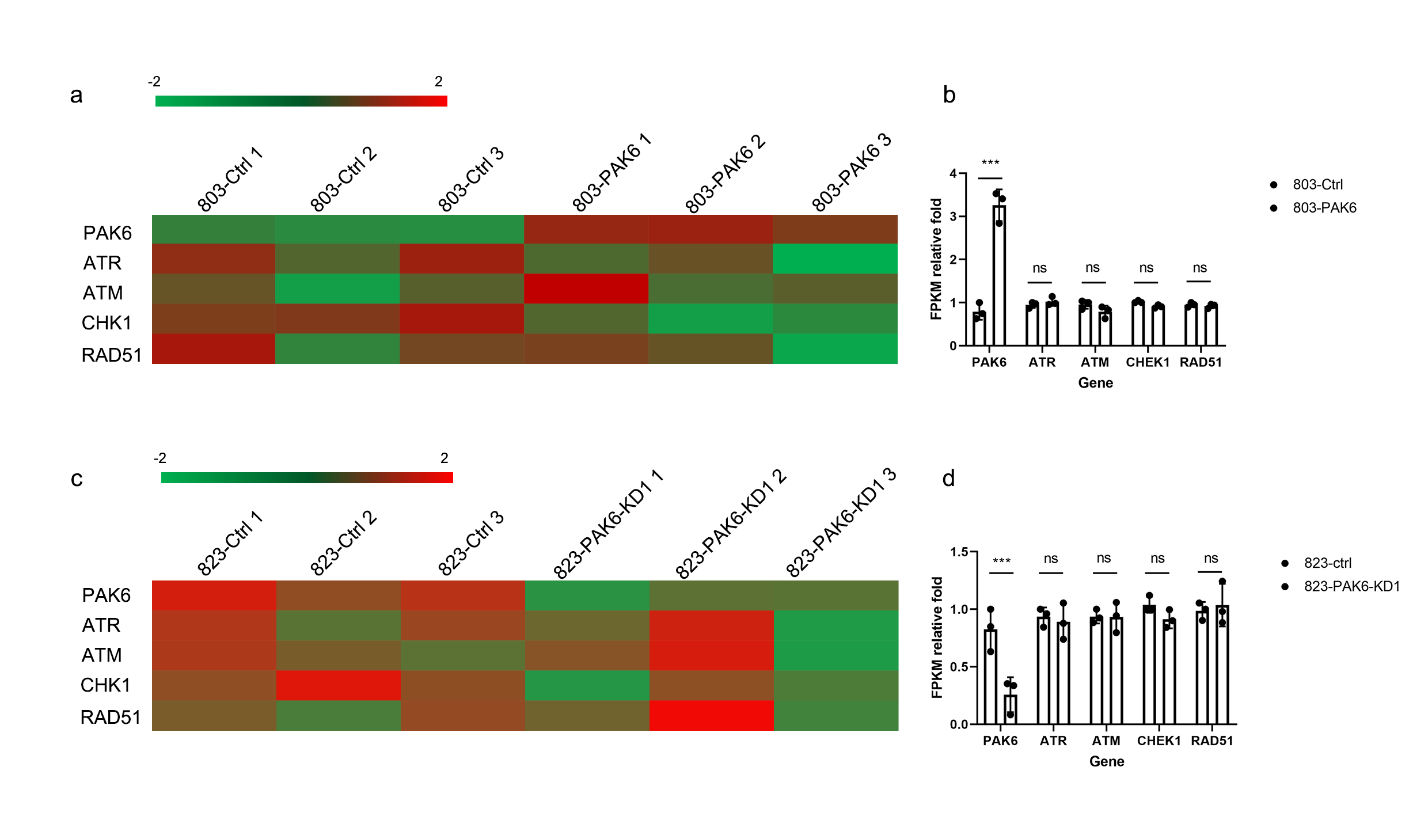


**Figure S5. Relationship between PAK6 and key DDR genes**. (a) Heat maps of targeted genes including PAK6, ATR, ATM, CHK1, and RAD51 in 803 GC cells line. (b) Comparison of relative expression (FPKM) of differentially expressed genes in 803 GC cells line. (c) Heat maps of targeted genes including PAK6, ATR, ATM, CHK1, and RAD51 in 823 GC cells line. (d) Comparison of relative expression (FPKM) of differentially expressed genes in 823 GC cells line. Data was replicated at least three times. ***: *P* < 0.001, ns: no statistical difference.


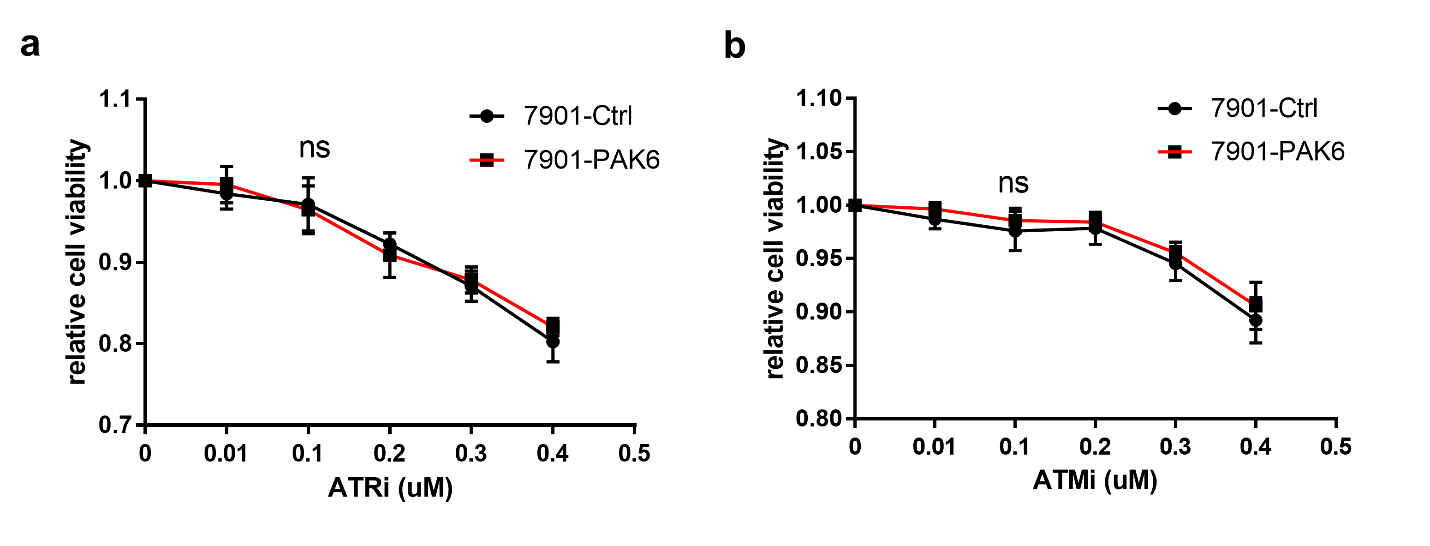


**Figure S6. The cytotoxicity of ATR and ATM inhibitors in GC cells.** (a) Dose-response curves of control cells or PAK6 overexpression cells after treated with ATR inhibitor AZD6738 for 24 hours. (b) Dose-response curves of control cells or PAK6 overexpression cells after treated with ATM inhibitor AZD0156 for 24 hours. Data presented as mean ± SD of at least three independent replicates. *: *P* < 0.05, ns: no statistical difference.


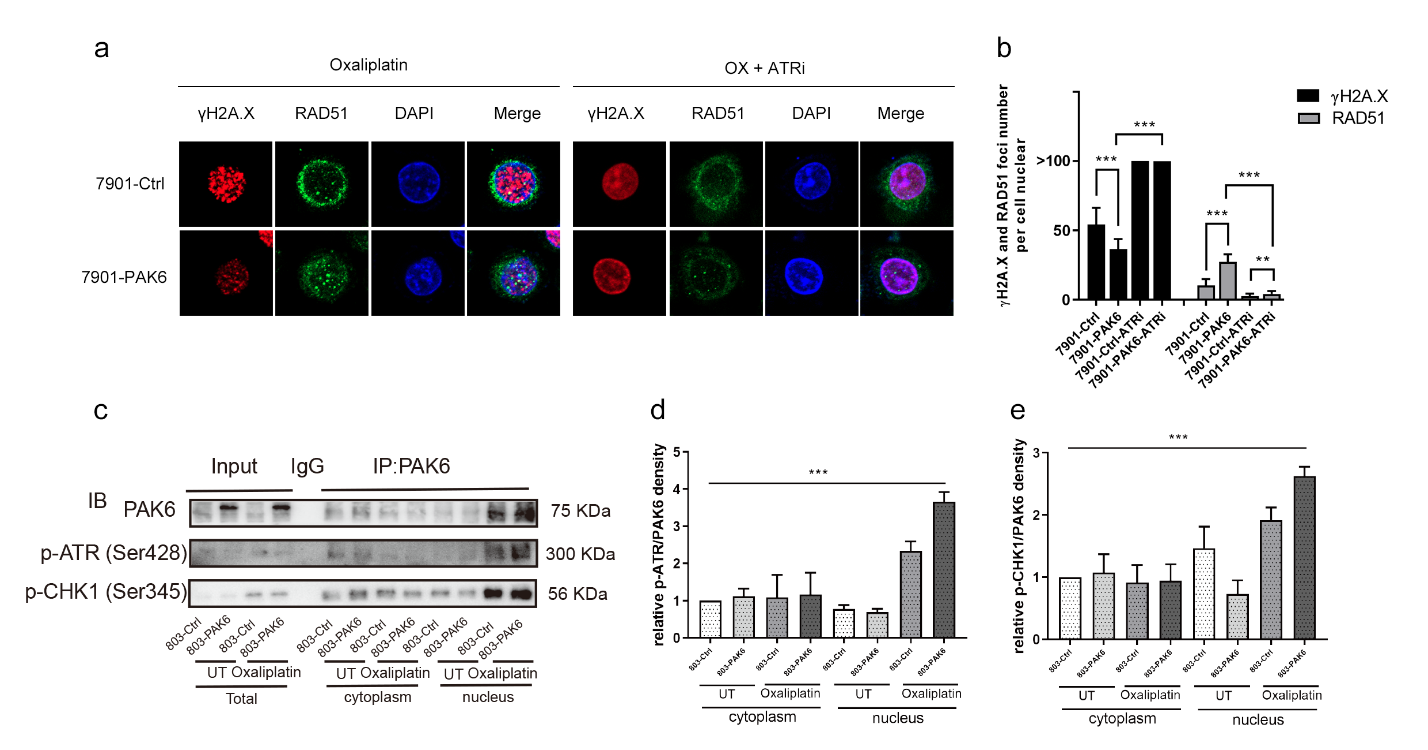


**Figure S7. ATR/CHK1 signaling activation is required for PAK6 mediated HR repair.** (a) Left: Representative images of γH2A.X staining (red), RAD51 staining (green), and DAPI staining (blue) of control cells and PAK6 overexpression cells in 7901 GC cell lines in the presence of oxaliplatin. Right: Representative images of γH2A.X staining (red), RAD51 staining (green), and DAPI staining (blue) of control cells and PAK6 overexpression cells in 7901 GC cell lines in the presence of combination of oxaliplatin and ATR inhibitor AZD6738. (b) Quantification of mean γH2A.X and RAD51 foci per cell. At least 30 cells per group were included for the counting and quantification. (c) Immunoprecipitation assay with anti-PAK6 antibody from control cells and PAK6 overexpression cells in 7901 GC cell line. Cells were untreated or subjected to oxaliplatin for 24 hours. And protein was lysed from cytoplasm and nucleus respectively. (d-e) Relative protein band intensity of p-ATR (d) and p-CHK1 (e) protein normalized against of PAK6. UT: untreated. ATRi: ATR inhibitor AZD6738. OX: oxaliplatin. **: *P* < 0.01, ***: *P* < 0.001.


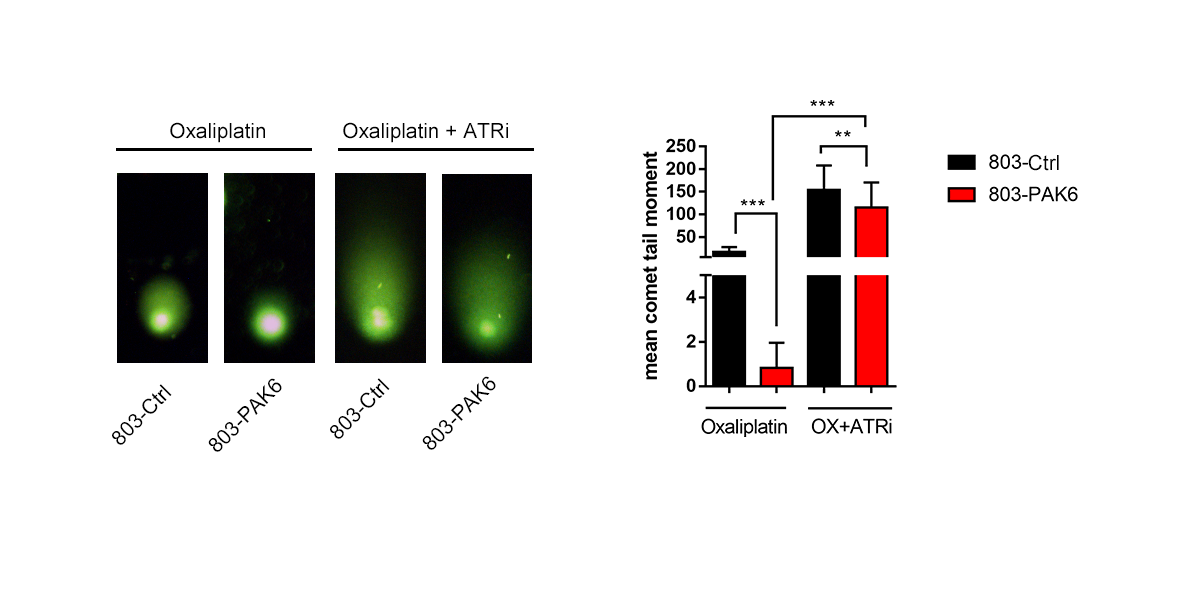


**Figure S8. ATR inhibitor AZD6738 could block PAK6 mediated HR repair in GC cells**. Left: Representative images of neutral comet assays of control cells and PAK6 overexpression cells in 803 GC cell lines in the presence of oxaliplatin or combination of ATR inhibitor AZD6738 and oxaliplatin; Right: Quantification of the percentages of DNA tail moments. At least 30 cells per group were included for the counting and quantification. ATRi: ATR inhibitor AZD6738. OX: oxaliplatin. **: *P* < 0.01, ***: *P* < 0.001.


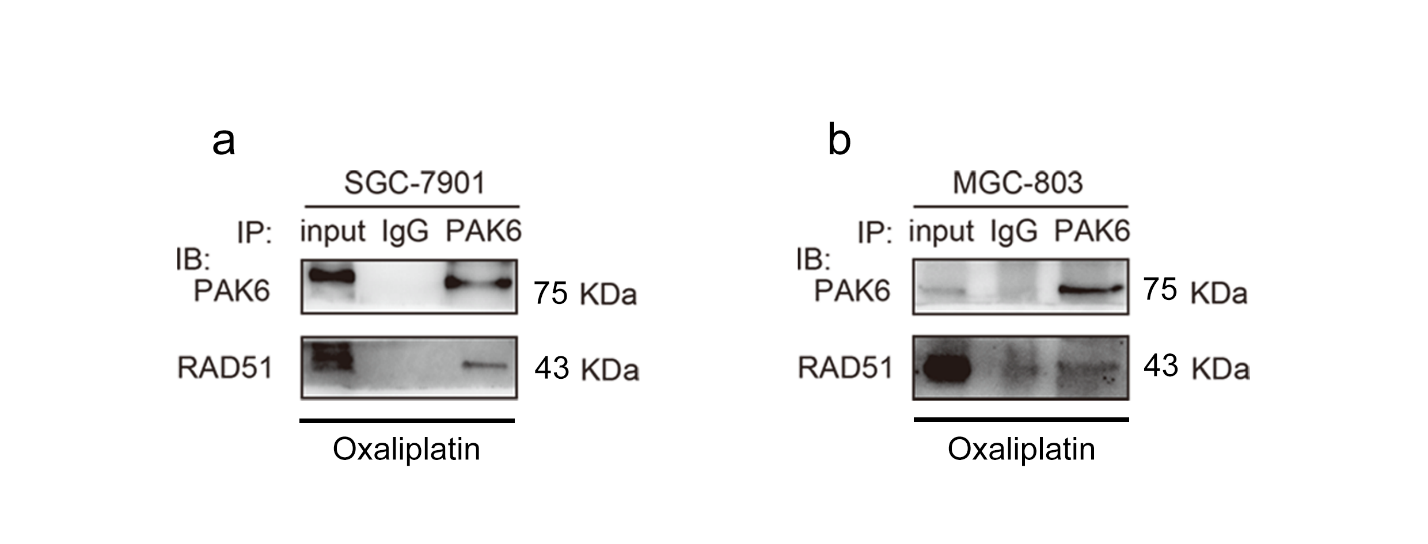


**Figure S9. Interaction of PAK6 and RAD51 in 7901 and 803 GC cells after exposure to oxaliplatin.**

**Supplementary Reference**

1. Jiang Y, Liu W, Li T, Hu Y, Chen S, Xi S*, et al.* Prognostic and Predictive Value of p21-activated Kinase 6 Associated Support Vector Machine Classifier in Gastric Cancer Treated by 5-fluorouracil/Oxaliplatin Chemotherapy. *EBioMedicine* 2017, **22:** 78-88.

2. Jiang Y, Zhang Q, Hu Y, Li T, Yu J, Zhao L*, et al.* ImmunoScore Signature: A Prognostic and Predictive Tool in Gastric Cancer. *Ann Surg* 2018, **267**(3)**:** 504-513.
