# Supplementary material for: PAK6 promotes homologous-recombination to enhance chemoresistance to oxaliplatin through ATR/CHK1 signaling in gastric cancer
Source: Cell Death Dis. 2022 Jul 28;13(7):658. doi: 10.1038/s41419-022-05118-8 (PMC9334622; doi:10.1038/s41419-022-05118-8)
Supplement: Supplementary file 3 — Original full length western blots [file 41419_2022_5118_MOESM3_ESM.docx]

Figure 2b



 PAK6-27-79-823-AGS-803



 GAPDH-27-79-823-AGS-803

Figure 2d



 823-nc kd1 kd2 pak6



 823 nc kd1 kd2 gapdh

Figure 2f



 ags pak6



 ags gapdh

Figure 2h



 79-803 pak6



 79-803 gapdh

Figure 2j



 803pak6



 803gapdh

Figure 4b



 EGFP ct pa



 gapdh ct pa ct pa ct pa ct pa



 Myc ISCEI ct pa



 PAK6 ct pa ct pa ct pa ct pa

Figure 4g



 823 atm



 823 atr



 823 c1



 823 cleaved 3



 823 gapdh



 823 h2ax



 823 pak6



 823 p-atm



 823 patr



 823 pc1



 823 rad51



 823 rh2ax



 AGS atm



 AGS atr



 AGS cleaved 3



 AGS gapdh



 AGS h2ax



 AGS pak6



 AGS patm



 AGS part



 AGS pc1



 AGS rad51



 AGS rh2ax

Figure 4h



 803 atm



 803 ATR



 803 CHK-1



 803 cleaved bcaspase3



 803 gapdh



 803 h2ax



 803 pak6



 803 p-atm



 803 p-atr1



 803 p-chk1



 803 rad51



 803 rh2ax



 7901 ATM



 7901 atr



 7901 C1



 7901 cleaved caspase3



 7901 gapdh



 7901 h2ax



 7901 PA



 7901 P-ATM



 7901 P-ATR



 7901 PC1



 7901 rad51



 7901 RH2AX

Figure 5a



 7901 53bp1



 7901 atm



 7901 ATR



 7901 c1



 7901 CASPACE3



 7901 gapdh



 7901 p-53bp1



 7901 pak6



 7901 p-atm



 7901 p-atr



 7901 PC1



 7901 rad51



 7901 rh2ax

Figure 5b



 803 53bp1



 803 atm



 803 atr



 803 c1



 803 clcaspase3



 803 gapdh



 803 p-53bp



 803 pak6



 803 p-atm



 803 p-atr



 803 p-c1



 803 rad51



 r-h2ax

Figure 5c



 7901 gapdh



 7901 lamin b1



 7901 pak6



 7901 rad51

Figure 5d



 803 gapdh



 803 lamin b1



 803 pak6



 803 rad51

Figure 5g



 7901 pak6



 7901 patr



 7901-pc1

Figure S6



 803 pak6


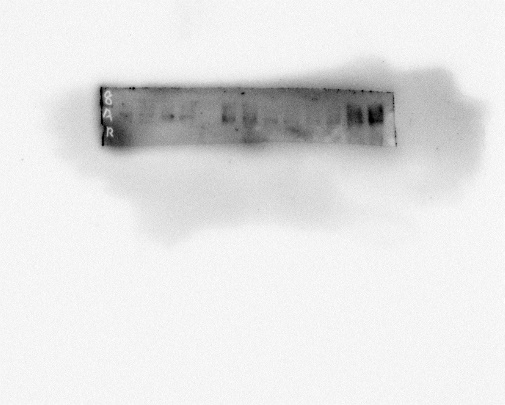
 803 patr

803 pc1

Figure S8

803 pak6

803 rad51

7901 pak6

7901 rad51
